# CHD8‐Dependent Chromatin Licensing Sustains Trophoblast Stem Cell Transcriptional Programs

**DOI:** 10.1002/advs.77823

**Published:** 2026-09-27

**Authors:** Yuanyuan Huang, Zhenhua Hu, Jiali Liao, Lijun Huang, Jinyu Liu, Chen Tu, Yuchen Li, Lulu Xie, Shilei Bi, Xiaobo Fang, Jingsi Chen, Lili Du, Wei Sun, Haibin Wang, Shuang Zhang, Dunjin Chen, Zhaowei Tu

**Affiliations:** ^1^ Department of Obstetrics and Gynecology Guangdong Provincial Key Laboratory of Major Obstetric Diseases Guangdong Provincial Clinical Research Center for Obstetrics and Gynecology Guangdong‐Hong Kong‐Macao Greater Bay Area Higher Education Joint Laboratory of Maternal‐Fetal Medicine The Third Affiliated Hospital of Guangzhou Medical University Guangzhou Guangdong China; ^2^ The Quzhou Affiliated Hospital Wenzhou Medical University Quzhou China; ^3^ Fujian Provincial Key Laboratory of Reproductive Health Research Department of Obstetrics and Gynecology The First Affiliated Hospital of Xiamen University School of Medicine Xiamen University Xiamen Fujian China

**Keywords:** CHD8, chromatin remodeling, placentation, trophoblast stem cells

## Abstract

Functional placentation relies on the precise balance between trophoblast stem cells (TSCs) self‐renewal and differentiation, yet how permissive chromatin states are maintained to support trophoblast gene regulatory networks (GRNs) remains poorly understood. Here, we show that CHD8‐dependent chromatin licensing is required for human and mouse TSCs maintenance. CHD8 depletion impairs cell‐cycle progression and self‐renewal and selectively downregulates key stemness‐associated genes. In vivo, reduced placental CHD8 expression is associated with recurrent pregnancy loss in humans, and trophoblast‐specific deletion of *Chd8* in mice disrupts placentation and causes miscarriage. Mechanistically, CHD8 maintains chromatin accessibility that supports the occupancy of key trophoblast transcription factors (TFs) at their target loci and is associated with KMT2A‐dependent H3K4me3 deposition at TSC self‐renewal genes. Increasing transcription factor abundance or global H3K4me3 levels is insufficient to restore transcription in CHD8‐depleted cells, indicating that chromatin accessibility constitutes a dominant regulatory constraint. Together, these findings identify CHD8‐dependent chromatin licensing as a critical regulatory layer that sustains TSC transcriptional programs and placental development.

## Graphical abstract

In this study, Huang et al., identify chromatin remodeler CHD8 as an essential factor in TSC maintenance and placental development. CHD8‐dependent chromatin accessibility supports the occupancy of key trophoblast TFs and is coupled to KMT2A‐associated H3K4me3 deposition at TSC stem and cell cycle genes, providing a permissive chromatin environment for active transcription that safeguards TSC self‐renewal.

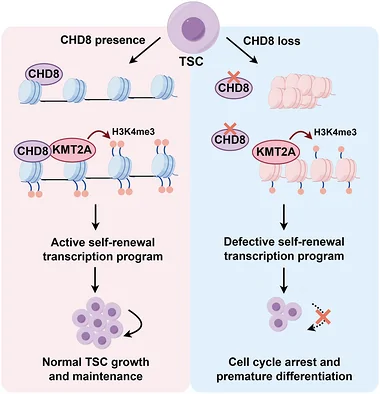

## 1 Introduction

Placental development is essential for successful mammalian reproduction, as it mediates nutrient exchange, hormonal signaling, and immune tolerance between the mother and the developing fetus. These functions critically depend on TSCs, which possess the capacity to differentiate into multiple trophoblast lineages while retaining the ability for self‐renewal [1]. Disruption of TSC maintenance or differentiation leads to placental insufficiency and adverse pregnancy outcomes, including miscarriage, preeclampsia, and fetal growth restriction [2]. Despite the importance of TSC integrity, the molecular mechanisms that sustain trophoblast transcriptional programs during early development remain incompletely understood.

Transcriptional regulation in stem cells relies not only on transcription factor availability but also on the permissive chromatin environments that enable factors binding and transcription activation. Studies employing animal models, TSCs, and cutting‐edge trophoblast organoids have pinpointed pivotal transcription factors (TFs) and signaling cascades that orchestrate TSC homeostasis [3, 4], including evolutionarily conserved GATA3, TEAD4, TFAP2C in both humans and mice [5, 6, 7, 8, 9, 10, 11, 12, 13]. However, the additional regulatory layers that are required to stabilize permissive chromatin states and support sustained transcriptional output in TSCs during placental development remains largely unknown.

ATP‐dependent chromatin remodeling factors utilize the energy from ATP hydrolysis to reconfigure nucleosome architecture or conformation, thereby modulating chromatin accessibility, and enabling TFs to occupy their cognate DNA binding sites [14]. These remodelers are grouped into four evolutionarily conserved families, including switch/sucrose nonfermentable (SWI/ SNF), imitation SWI (ISWI), INO80/SWI2/SNF2 related (SWR), and CHD (chromodomain helicase DNA‐binding) [15]. Recent studies highlight the essential roles of SWI/SNF complexes in human and murine TE lineage maintenance and STB differentiation, functioning as “licensing” factors that establish or maintain a permissive chromatin landscape [7, 16, 17, 18]. Whether other chromatin licensing factors operate in TSCs, and how they contribute to placental development and function in vivo, remains unclear.

CHD8 is an ATP‐dependent chromatin remodeler of CHD family that has been implicated in transcriptional regulation, chromatin organization, and developmental processes across multiple contexts [19]. Genetic studies have linked CHD8 functions to neurodevelopment, hematopoiesis, and intestinal homeostasis [20, 21, 22, 23, 24, 25, 26, 27]. At the molecular level, CHD8 has been found as a negative regulator of P53 and β‐catenin pathway [27, 28], as well as associating with active promoters and enhancers and to interact with components of transcriptional and epigenetic machinery [29, 30]. However, whether CHD8 plays a direct role in maintaining chromatin accessibility and transcriptional competence in extraembryonic lineages has not been explored.

Here, we investigated the role of CHD8 in TSC regulation and placental development. Using human and mouse TSCs, together with a trophoblast‐specific *Chd8* knockout mouse model, we show that CHD8 maintains chromatin accessibility and transcriptional programs required for TSC maintenance. CHD8‐dependent chromatin accessibility supports the occupancy of key trophoblast TFs and is coupled to KMT2A‐associated H3K4me3 deposition at trophoblast self‐renewal genes. These findings identify CHD8‐dependent chromatin licensing as a critical regulatory layer that safeguards TSC function and placental development.

## 2 Results

### 2.1 CHD8 Preferentially Associates With Accessible Chromatin in hTSCs

**FIGURE 1:**
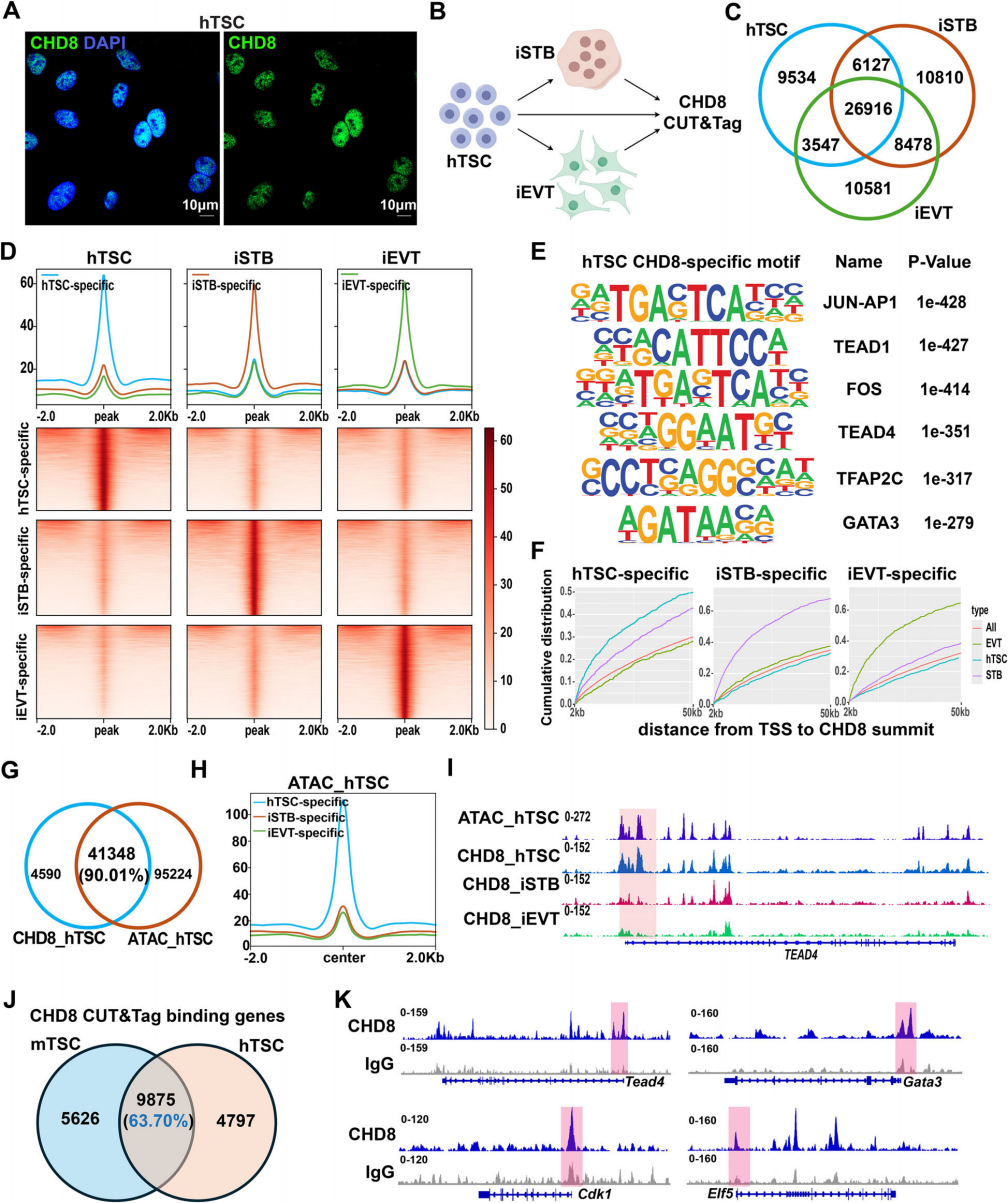
CHD8 preferentially associates with accessible chromatin in hTSCs. (A) Immunofluorescence staining of CHD8 in hTSCs derived from first‐trimester CTBs. Scale bars, 10 µm. CTB, cytotrophoblast. (B) Schematic representation of CHD8 chromatin profiling in hTSCs, iSTBs and iEVTs. (C) Venn diagram showing the overlap among hTSC, iEVT, and iSTB CHD8 peaks. (D) Metaplots and heatmaps showing enrichment of CHD8 signals at hTSC‐specific, iSTB‐specific, and iEVT‐specific CHD8 peaks. (E) Motif enrichment of hTSC‐specific CHD8 peaks showing the top binding motifs. P‐values were calculated from cumulative hypergeometric distribution using the Homer2 package. (F) The cumulative distributions of hTSC‐specific, iEVT‐specific, iSTB‐specific peaks, and all genes showing the fraction of genes within defined distances (x axis) between their transcription start sites (TSSs) to the nearest hTSC‐specific (left panel), iSTB‐specific (middle panel), and iEVT‐specific (right panel) CHD8 peaks. (G) Venn diagram showing the overlap between CHD8 peaks and ATAC‐seq peaks in hTSC cells. (H) Metaplot showing the enrichment of hTSC ATAC‐seq signals across hTSC‐specific, iSTB‐specific, and iEVT‐specific CHD8 peaks. (I) IGV tracks displaying the enrichment of ATAC‐seq and CHD8 occupancy signals at *TEAD4* gene loci in trophoblasts. (J) Venn diagram comparing CHD8‐associated genes identified by CUT&Tag in mTSCs and hTSCs. (K) IGV tracks of CHD8 peaks at *Tead4, Gata3, Cdk1*, and *Elf5* gene loci in mTSCs.

We established a human TSC (hTSC) model as previously described [31]. The cells robustly expressed stem‐cell marker *TEAD4* and efficiently differentiated into STBs expressing *CGB* and *SDC1* or extravillous trophoblasts (EVTs) expressing *HLA‐G* and *MMP2* (Figure S1A,B). To investigate the potential roles of CHD8, we first detected its expression hTSCs. Immunostaining revealed abundant CHD8 in hTSCs derived from cytotrophoblasts (CTBs; Figure 1A), and its expression remained unchanged during differentiation (Figure S1C). We then performed CUT&Tag in hTSCs and in vitro differentiated STBs (iSTBs) and EVTs (iEVTs) to map dynamic CHD8 occupancy (Figure 1B). CHD8 bound predominantly to distal intergenic, intronic, and promoter regions (Figure S1D). Differential peak analysis identified 9534, 10 810, and 10 581 lineage‐specific CHD8 sites in hTSC, iSTBs, and iEVTs, respectively (Figure 1C, D). Motif analysis of hTSC‐specific CHD8 peaks revealed enrichment of TEAD, TFAP2C, and GATA3 binding signatures (Figure 1E).

To relate lineage‐specific CHD8 to gene expression, we integrated our CUT&Tag data with published transcriptomes of hTSC, STBs, and EVTs [32]. hTSC‐specific CHD8 occupancy was preferentially associated with hTSC signature genes, whereas iSTB‐ and iEVT‐specific peaks were associated with transcripts enriched in the corresponding lineages (Figure 1F). KEGG analysis implicated hTSC‐specific CHD8 bindings in Hippo signaling regulation (Figure S1E). hTSC‐specific CHD8 peaks also showed partial alignment with iSTB lineage transcripts (Figure 1F). To examine this relationship further, we operationally classified STB genes as early or late according to their expression in hTSCs (FPKM≥1 and FPKM<1, respectively). CHD8 occupancy was associated with hTSC and early‐STB genes but not with EVT lineage genes (Figure S1F,G). Moreover, hTSC‐specific CHD8 peaks partially overlapped GATA3, MSX2, and ARID1A binding sites and H3K27ac‐enriched regions, which are related to STB differentiation (Figure S1H). Thus, CHD8 occupancy is preferentially associated with the hTSC stem‐state program and a subset of early STB‐associated transcriptional signatures.

To examine the relationship between CHD8 occupancy and active chromatin, we performed ATAC‐seq in hTSCs. Most CHD8‐binding sites (90.01%) overlapped accessible chromatin (Figure 1G), and hTSC‐specific CHD8 peaks exhibited stronger ATAC‐seq signals than iSTB‐ or iEVT‐specific peaks (Figure 1H, Figure S1I). The IGV tracks at the hTSC markers *CDH1* and *TEAD4* confirmed these observations, CHD8 binding at the promoters of stem markers decreased during differentiation, whereas it increased at the *SDC1* in iSTBs and *HLA‐G* in iEVTs (Figure 1I, Figure S1J).

We next performed CHD8 CUT&Tag in mouse TSCs (mTSCs). Of the genes associated with CHD8 peaks in mTSCs, 63.7% were also identified as CHD8‐associated genes in hTSCs (Figure 1J). Shared loci included the representative genes such as key TSC transcription factors *Tead4*, *Gata3*, and *Elf5*, and the cell‐cycle gene *Cdk1* (Figure 1K). Together, these findings identify CHD8 as a candidate chromatin regulator of conserved TSC transcriptional programs.

### 2.2 CHD8 Is Required for Trophoblast Stem Cell Self‐Renewal

**FIGURE 2:**
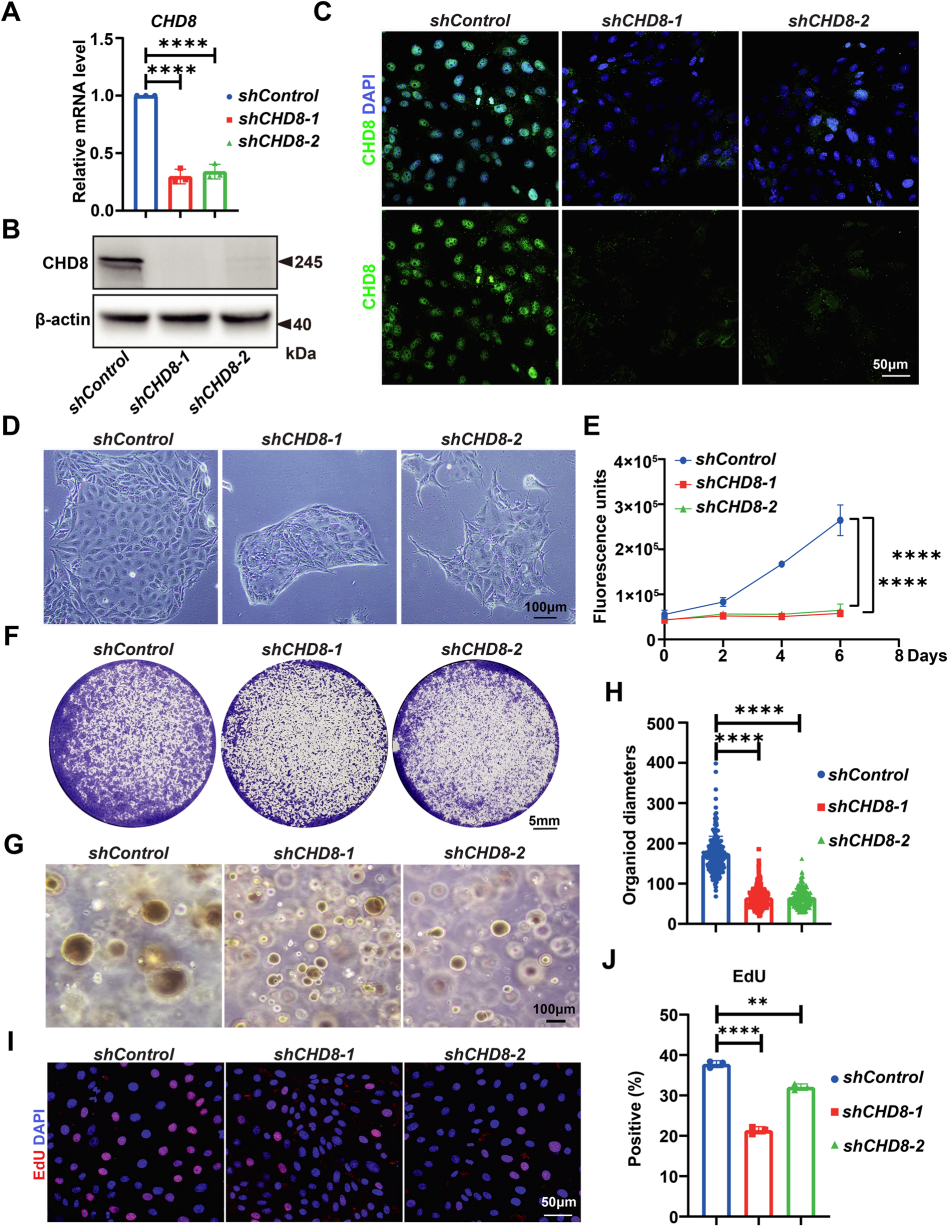
CHD8 depletion impairs human TSC self‐renewal. (A) Relative *CHD8* mRNA expression in *shControl* and *shCHD8* hTSCs at 72 h after transfection. The data represent three biological replicates. ****p < 0.0001. (B) Western blot confirmation of CHD8 protein deletion in *shCHD8* hTSCs at 72 h after infection. β‐actin was used as the loading control. (C) Representative CHD8 immunostaining pictures in *shControl* and *shCHD8* hTSCs. Scale bar, 50 µm. (D) Phase contrast images of *shControl* and *shCHD8* hTSCs at 72 h after knockdown. Scale bar, 100 µm. (E) PrestoBlue Cell Viability analysis of hTSCs growth in control and *CHD8* depleted group (*n* = 3 per group), ****p < 0.0001. (F) Colony forming pictures of *shControl* and *shCHD8* hTSCs. Scale bar, 5 mm. (G) Micrographs of organoid formed from *shControl* and *shCHD8* hTSCs. Scale bar, 100 µm. (H) The statistics of organoid diameters of *shControl* and *shCHD8* hTSCs. ****p < 0.0001. (I) EdU immunostaining to detect the cell proliferation after CHD8 knockdown. Scale bar, 50 µm. (J) The percentages of EdU^+^ hTSCs in *shControl* and *shCHD8* groups. Biological replicates, *n* = 3. **p < 0.01, ****p < 0.0001.

To determine whether CHD8 is required for hTSC function, we used two short‐hairpin RNAs (shRNAs) and achieved efficient knockdown of CHD8 mRNA and protein after 72 h of infection in hTSCs (Figure S2A, Figure 2A–C). CHD8 depletion disrupted colony morphology, reduced cell growth under hTSC culture condition, and decreased colony‐forming efficiency (Figure 2D–F). It also markedly impaired organoid formation and reduced organoid size (Figure 2G,H). Next, we examined the proliferation of hTSCs using both EdU incorporation assay and Ki67 staining. The percentages of EdU^+^ and Ki67^+^ hTSCs were significantly decreased after CHD8 knockdown (Figure 2I,J, Figure S2B,C), demonstrating that CHD8 is essential for hTSC self‐renewal. Although our previous study showed that CHD8 suppresses p53‐mediated apoptosis [27], analyses of cleaved caspase‐3 and p53 signaling revealed no significant increase in cell death after CHD8 depletion in hTSCs (Figure S2D–F). The above findings proved CHD8 as an essential regulator of hTSC self‐renewal.

**FIGURE 3:**
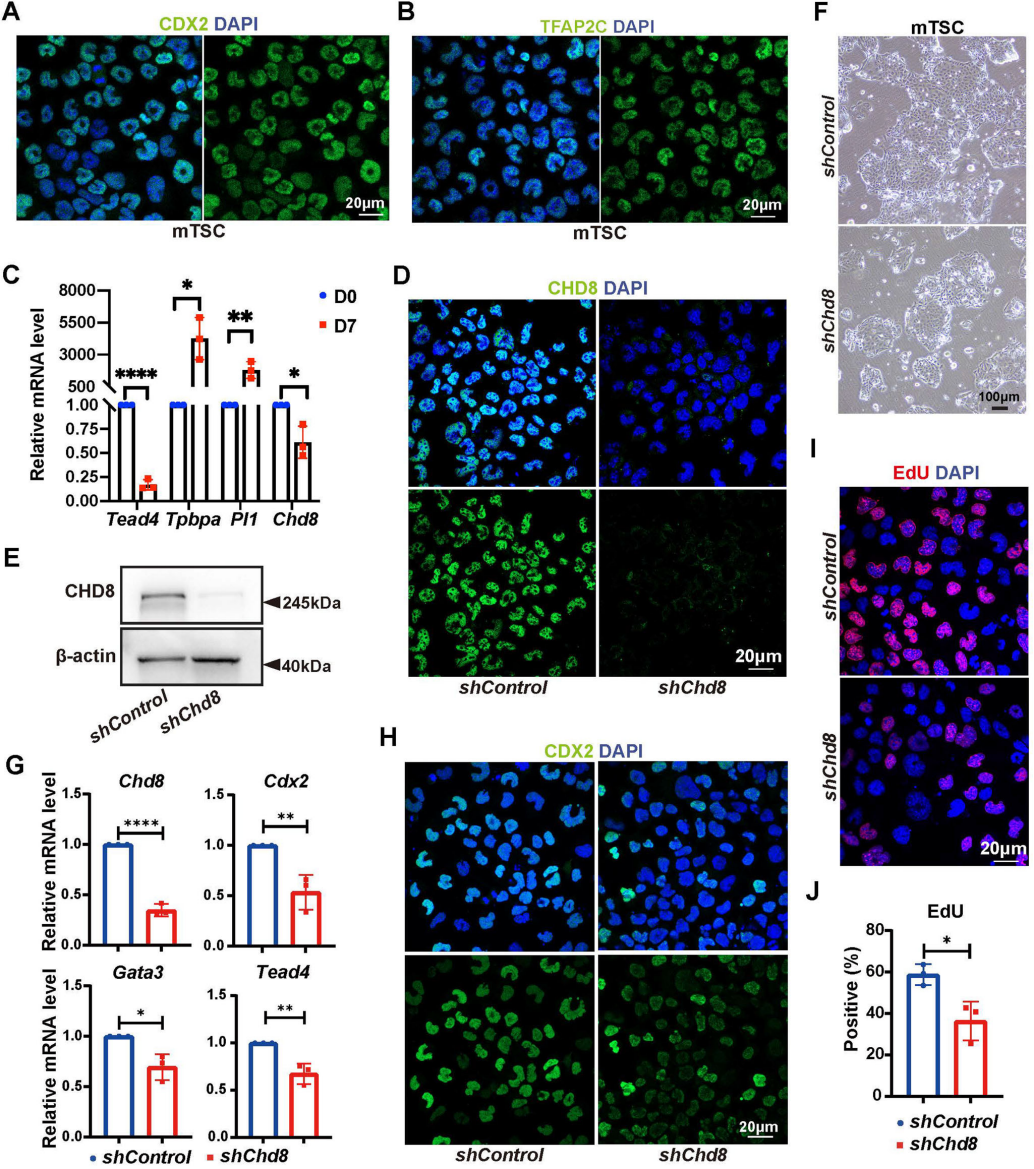
CHD8 is required for mTSC maintenance. (A, B) Immunofluorescence staining of CDX2 (A) and TFAP2C (B) in mTSCs. Scale bars, 20 µm. (C) Relative expression of *Tead4*, *Tpbpa*, *Pl1*, and *Chd8* in mouse TSCs at day 0 (D0, blue) and day 7 (D7, red) upon differentiation. Biological replicates, *n* = 3. *p < 0.05, **p < 0.01, ****p < 0.0001. (D) Representative images of CHD8 staining in *shControl* and *shChd8* mTSCs. Scale bar, 20 µm. (E) Western blots confirmation of CHD8 depletion in *shChd8* mTSCs. β‐actin was used as the loading control. (F) Phase‐contrast images of *shControl* and *shChd8* mTSCs at 48 h after lentiviral infection. Scale bar, 100 µm. (G) Relative *Chd8*, *Cdx2*, *Gata3*, and *Tead4* mRNA expression in *shControl* and *shChd8* mTSCs. Biological replicates, *n* = 3. *p < 0.05, **p < 0.01, ****p < 0.0001. (H) Representative CDX2 immunostaining pictures in *shControl* and *shChd8* mTSCs. Scale bar, 20 µm. (I) EdU immunostaining to detect the cell proliferation after *Chd8* knockdown in mTSCs. Scale bar, 20 µm. (J) The percentages of EdU^+^ mTSCs in *shControl* and *shChd8* groups. Biological replicates, *n* = 3. *p < 0.05.

To determine whether this function is conserved in mice, we used mTSCs established in our previous study [33]. These cells expressed CDX2 and TFAP2C and retained the capacity to differentiate (Figure 3A–C). CHD8 was also abundant in mTSCs and decreased modestly during differentiation (Figure 3C). Depletion of *Chd8* by shRNA dramatically reduced the CHD8 protein level in mTSCs after 72 h of transfection (Figure 3D,E). *Chd8*‐depleted mTSCs showed impaired cell growth and disrupted colony morphology (Figure 3F), reduced expression of *Cdx2*, *Gata3* and *Tead4* (Figure 3G,H), as well as blocked cell cycle indicated by EdU incorporation assay (Figure 3I,J), resembling the phenotypes observed in hTSCs. Thus, CHD8 is also required for mTSC maintenance.

### 2.3 *Chd8* Loss in Trophoblast Disrupts Placental Development and Causes Miscarriage In Vivo

**FIGURE 4:**
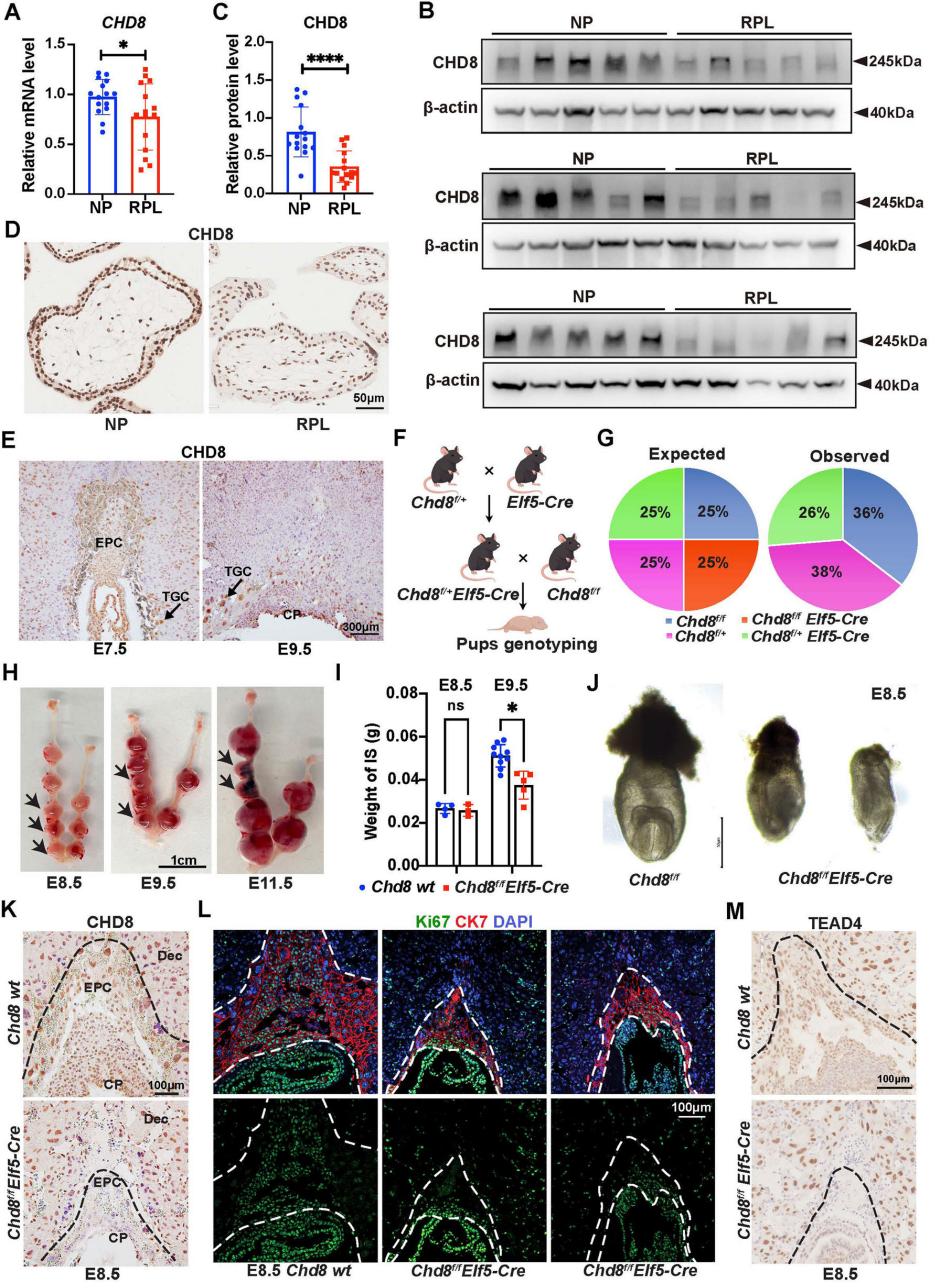
CHD8 in trophoblast progenitors is required for placental development in vivo. (A) Relative *CHD8* mRNA expression in the placenta villi from terminated normal pregnancy (NP) and recurrent pregnancy loss (RPL). *p < 0.05. *n* = 15. (B) Western blots detection of CHD8 protein in NP and RPL villus. β‐actin was used as the loading control. *n* = 15. (C) The quantification of CHD8 protein level in NP and RPL villi detected by immunoblots. ****p < 0.0001. (D) Immunochemistry of CHD8 in NP and RPL placenta villi. Scale bar, 50 µm. (E) Immunochemistry of CHD8 in wild type placenta at E7.5 and E9.5. EPC, ectoplacental cone. TGC, trophoblast giant cell. CP, chorion plate. Scale bar, 300 µm. (F) Strategy of generating *Chd8* trophoblast‐specific knockout mouse model. (G) Pie charts showing the proportion of live offsprings from trophoblast specific CHD8 deletion mice. 95 pups were included in total. (H) Implantation sites (IS) in female *Chd8^f/f^* mice crossed with male *Chd8^f/+^Elf5‐Cre* mice at E8.5, E9.5, and E11.5. The black arrows indicate *Chd8^f/f^Elf5‐Cre* embryos. Scale bar, 1 cm. (I) Weight of ISs with wild type and *Chd8^f/f^Elf5‐Cre* embryos. *p < 0.05, ns indicates not significant. (J) Bright field images of *Chd8^f/f^* and *Chd8^f/f^Elf5‐Cre* embryos isolated from E8.5 ISs. Scale bar, 50 µm. (K) Immunochemistry of CHD8 in *Chd8 wild type* and *Chd8^f/f^Elf5‐Cre* ISs harvested at E8.5. EPC, ectoplacental cone. CP, chorion plate. Dec, decidual cells. Scale bar, 100 µm. (L) Immunostaining of Ki67 (green) and CK7 (red) in the trophoblast of *Chd8 wild type* and *Chd8^f/f^Elf5‐Cre* embryos at E8.5. Scale bar, 100 µm. (M) Immunochemistry of TEAD4 in the trophoblast of *Chd8 wild type* and *Chd8^f/f^Elf5‐Cre* embryos at E8.5. Scale bar, 100 µm.

To explore the clinical relevance of CHD8, we quantified its mRNA and protein abundance in first‐trimester villous tissues from electively terminated healthy pregnancies (NP) and unexplained recurrent pregnancy loss (RPL). CHD8 was reduced in RPL samples (Figure 4A–C), with reduced staining in both CTBs and STBs of the villi (Figure 4D). These findings establish an association between reduced placental CHD8 expression and RPL but do not determine whether reduced CHD8 is a cause or a consequence of pregnancy loss.

To test the role of trophoblast‐intrinsic CHD8 in placental development and function, we used mouse model to trace the effects of CHD8 deletion on placental development. CHD8 immunostaining at E7.5, E9.5, and E11.5 revealed its robust expression in trophoblast progenitors and differentiated trophoblasts (Figure 4E; Figure S3A). We then established CHD8 trophoblast‐specific deletion by crossing *Chd8^flox/flox^* with *Elf5‐Cre* mice (Figure 4F). No offsprings with *Chd8^f/f^Elf5‐Cre* genotype were recovered, indicating the trophoblast deletion of *Chd8* causes embryonic lethality (Figure 4G). Implantation sites were grossly comparable with controls before E8.5, whereas mutant embryos exhibited pronounced growth retardation and placenta defects by E9.5, and were completely resorbed by E11.5 (Figure 4H,I).

At E8.5, CHD8 was undetectable in trophoblasts of dissected *Chd8^f/f^Elf5‐Cre* embryos, and which were smaller than control conceptuses and showed visibly impaired ectoplacental cone (EPC) formation (Figure 4J,K, Figure S3B). Consistent with TSC phenotypes, the number of Ki67^+^CK7^+^ trophoblasts in *Chd8^f/f^Elf5‐Cre* EPCs was significantly reduced, whereas the apoptosis in trophoblasts was not altered (Figure 4L, Figure S3C). At E9.5, histological examination further confirmed failed placentation after CHD8 deletion (Figure S3D). Moreover, immunostaining results showed that TEAD4 level was reduced in CHD8‐deleted EPCs (Figure 4M, Figure S3E). Together, these findings demonstrate that trophoblast CHD8 is required for placental development and function in vivo.

### 2.4 CHD8 Deletion Leads to Aberrant Transcription Programs in hTSCs

**FIGURE 5:**
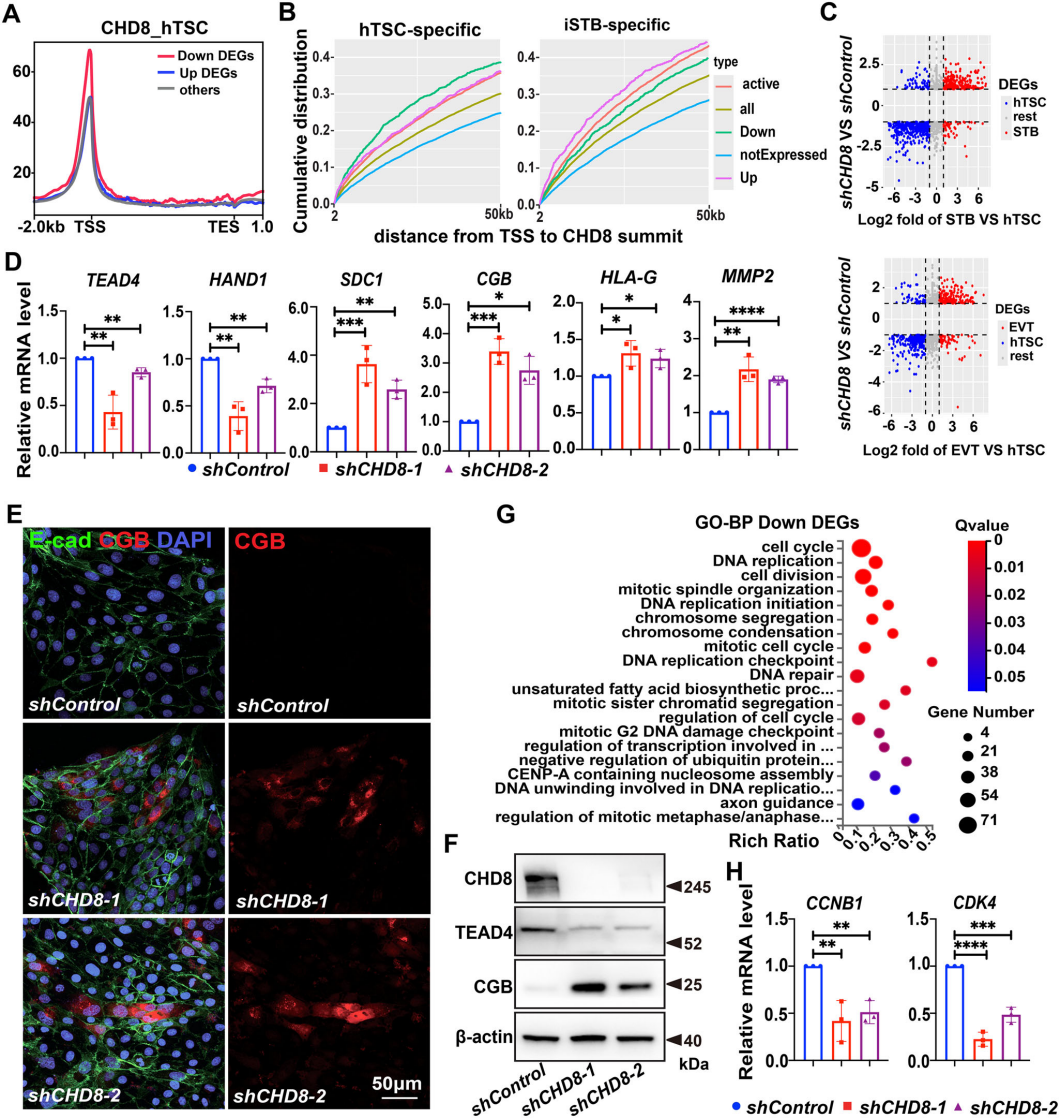
CHD8 deletion disrupts hTSC transcriptional programs. (A) Metaplots of CHD8 signal at down‐regulated, up‐regulated, and not differentially expressed (others) genes upon CHD8 depletion in hTSCs (*shCHD8* vs. *shControl*). (B) The cumulative distributions of genes that are actively expressed (active; FPKM≥5), not expressed (FPKM < 1) in hTSCs, that are down‐regulated (Down), up‐regulated (Up) upon CHD8 depletion, and all genes (all) showing the fraction of genes within defined distances (x‐axis) between their transcription start sites (TSSs) to the nearest hTSC‐specific (left panel) and STB‐specific (right panel) CHD8 peaks. (C) Scatter plots comparing gene expression changes between STB vs. hTSC (up panel) and EVT vs. hTSC (down panel), and between the CHD8 depletion (*shCHD8*) vs. *shControl* hTSC. hTSC‐ and EVT/STB‐enriched genes were colored in blue and red, respectively. (D) RT‐qPCR of stem, EVT, and STB markers in *shControl* and *shCHD8* hTSCs. The data represent 3 biological replicates. *p < 0.05, **p < 0.01, ***p < 0.001, ****p < 0.0001. (E) Representative images of E‐cadherin and CGB immunostaining in *shControl* and *shCHD8* hTSCs at 72 h after knockdown. Scale bar, 50 µm. (F) Western blot detection of CHD8, TEAD4, and CGB in *shControl* and *shCHD8* hTSCs. β‐actin was used as the loading control. (G) Functional enrichment analysis of down‐regulated genes in the *shCHD8* vs. *shControl* hTSCs showing significantly inhibited biological processes. (H) Relative expression of cell cycle genes in *shControl* and *shCHD8* hTSCs. The data represent 3 biological replicates. **p < 0.01, ***p < 0.001, ****p < 0.0001.

To obtain a genome‐wide view of transcriptional changes after CHD8 depletion, we performed RNA‐seq in *shControl* and *shCHD8* hTSCs. Biological triplicates from each group were highly correlated (Figure S4A), and differential expression analysis identified 504 up‐regulated and 716 down‐regulated genes (cutoff: |log2FC|≥1, Q value<0.05; Figure S4B). Notably, CHD8 occupancy was strongest at the promoters of down‐regulated genes when compared to up‐regulated and unchanged genes (Figure 5A). hTSC‐specific CHD8 peaks were preferentially associated with down‐regulated genes, whereas iSTB‐ and iEVT‐specific peaks were aligned with up‐regulated genes (Figure 5B, Figure S4C). Integration with published hTSC, iSTB, iEVT transcriptomes further showed that CHD8 depletion preferentially silenced hTSC signature genes while increasing subsets of iSTB‐ and iEVT‐enriched transcripts (Figure 5C, Figure S4D). RT‐qPCR confirmed reduced expression of the stemness regulator *TEAD4* and hTSC growth factor *HAND1* [32], but not *GATA3* and *TP63* (Figure 5D, Figure S4E). STB markers *SDC1* and *CGB* and EVT markers *HLA‐G and MMP2* were up‐regulated (Figure 5D). Decreased TEAD4 and increased CGB and MMP2 expression in CHD8 depleted cells were further confirmed at protein level (Figure 5E,F, Figure S4F). Besides, in consistent with the blocked cell proliferation in CHD8 depleted hTSCs, the down‐regulated genes were mostly enriched in Cell Cycle term in Gene Ontology (GO) analysis (Figure 5G), whereas up‐regulated genes were associated in cell‐matrix adhesion and inflammatory responses (Figure S4G). The decreased expression of cell cycle genes, including *CCNB1*, *CDK4*, and *CDK1*, was further confirmed by RT‐qPCR (Figure 5H, Figure S4E).

To assess whether the defective cell cycle genes expression was directly regulated by CHD8 or were secondary to differentiation due to stem‐state loss, we stained the control and CHD8 depleted hTSC with CGB and EdU, and examined the cell cycle in CGB negative hTSCs. Consistent with our previous observation, we detected significantly decreased CGB^−^EdU^+^ hTSCs after CHD8 depletion (Figure S5), suggesting that cell cycle defects in CHD8 deficient hTSCs were not majorly caused by premature STB differentiation.

In addition, to test the reproducibility of these findings, we performed CHD8 knockdown in another two independent human TSC lines derived from the first‐trimester placenta villi. Both lines showed impaired maintenance and proliferation together with altered expression of key TSC markers, consistent with the phenotypes observed in the primary hTSC line used throughout this study (Figure S6). Together, these results show that CHD8 supports stemness‐ and cell‐cycle‐associated transcriptional programs in hTSCs.

### 2.5 Loss of CHD8 in hTSCs Leads to Selective Collapse of Chromatin Accessibility at Key Transcription Factors’ Regulatory Elements

**FIGURE 6:**
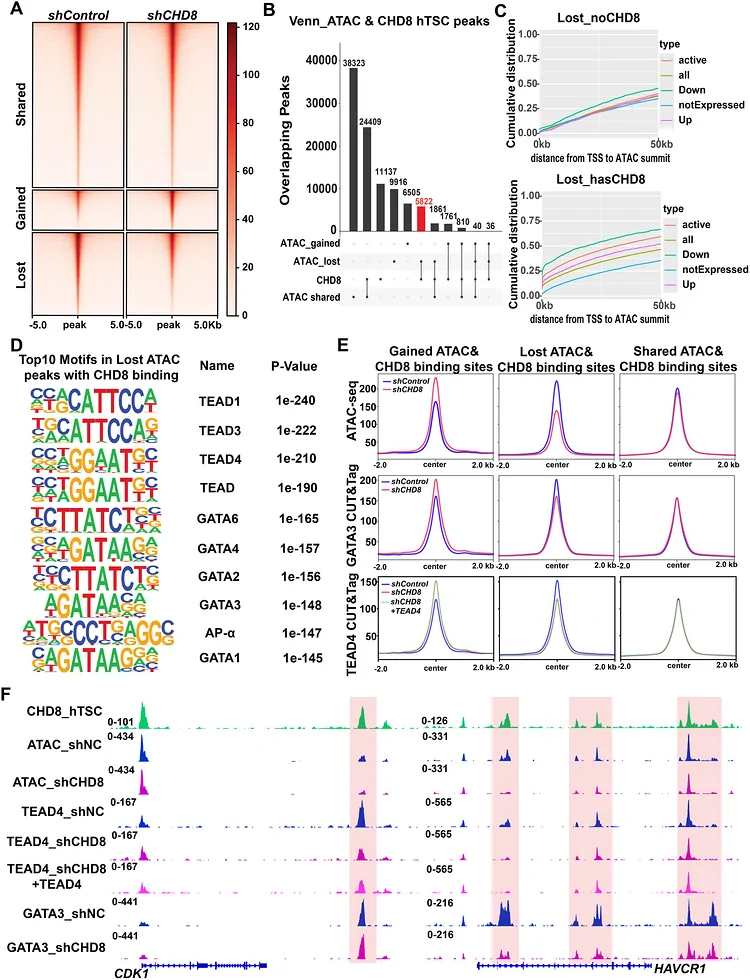
Loss of CHD8 leads to selective collapse of chromatin accessibility at TEAD4 and GATA3 regulatory elements. (A) Heatmap showing the enrichment of ATAC‐seq signals at shared, gained, and lost ATAC‐seq peaks upon CHD8 depletion in hTSCs. (B) Upset plot depicting the overlap between CHD8 peaks and the shared, gained, and lost ATAC‐seq peaks in hTSCs. (C) The cumulative distributions of genes that are actively expressed (active; FPKM≥5), not expressed (FPKM<1) in hTSCs, that are down‐regulated (Down), up‐regulated (Up) upon CHD8 depletion, and all genes (all) showing the fraction of genes within defined distances (x‐axis) between their transcription start sites (TSSs) to the nearest lost ATAC‐seq peaks that overlap CHD8 binding sites (Lost_hasCHD8) and that do not overlap CHD8 binding sites (Lost_noCHD8). (D) The top 10 enriched motifs identified from the lost ATAC‐seq peaks upon CHD8 depletion that overlap CHD8 binding sites in hTSCs. P‐values were calculated from cumulative hypergeometric distribution using the Homer2 package. (E) Metaplots showing the enrichment of ATAC‐seq, GATA3 CUT&Tag, TEAD4 CUT&Tag signals in *shControl, shCHD8, shCHD8* with TEAD4 overexpression hTSC cells at gained (left panel), lost (middle panel), and shared (right panel) ATAC‐seq peaks that overlap with CHD8 binding sites upon CHD8 depletion. (F) IGV tracks displaying the enrichment of ATAC‐seq signal, TEAD4, GATA3, and CHD8 occupancy at *CDK1* and *HAVCR1* gene loci in *shControl* and *shCHD8* hTSCs.

To determine whether CHD8 maintains chromatin accessibility in TSCs, we performed ATAC‐seq after CHD8 depletion in hTSCs. We identified 20865 gained and 43039 lost peaks in CHD8 depleted hTSCs (Figure 6A), with modest changes in their overall peak genomic distribution (Figure S7A). Motif enrichment revealed JUN‐AP1 signatures within gained peaks, whereas TEAD, GATA, and CTCF motifs for lost peaks (Figure S7B,C). Regions that gained accessibility after CHD8 depletion were preferentially associated with upregulated genes and STB‐lineage genes, whereas regions that lost accessibility were enriched near downregulated genes and hTSC‐lineage genes (Figure S7D,E).

To elucidate CHD8's direct role in governing chromatin accessibility, we integrated CHD8 CUT&Tag with ATAC‐seq data. Co‐occupancy analysis revealed 5822 lost ATAC peaks overlapping CHD8 binding, versus only 1761 gained peaks (Figure 6B). Lost CHD8‐bound accessible regions were preferentially associated with genes downregulated in *shCHD8* hTSCs (Figure 6C, Figure S7F). Motif analysis showed enrichment of TEAD and GATA TFs in lost CHD8‐bound peaks, whereas BATF, ATF3, JUN, AP‐1 motifs for gained ones (Figure 6D, Figure S7G). To validate the Homer predictions, we conducted GATA3 and TEAD4 CUT&Tag in control and CHD8‐depleted hTSCs (Figure S7H,I). Bindings of both TFs was markedly reduced at lost CHD8‐bound sites (Figure 6E). Importantly, TEAD4 overexpression in *shCHD8* cells did not restore its occupancy at these sites (Figure 6E), suggesting that chromatin closure but not reduced TEAD4 abundance is the primary barrier of its reduced binding. *CDK1* and *HAVCR1*, established TEAD4 and GATA3 targets, respectively [11, 34], both displayed diminished their TF occupancy, respectively (Figure 6F). These findings demonstrated that CHD8‐dependent chromatin accessibility constitutes a dominant regulatory constraint on transcription factor function in trophoblast stem cells. CHD8 licenses regulatory regions to remain accessible, thereby enabling productive transcription factor engagement. In the absence of this licensing layer, transcription factor activity cannot be restored by increased factor availability alone.

### 2.6 CHD8 Is Associated With KMT2A‐Dependent H3K4me3 Deposition at Key hTSC Genes

**FIGURE 7:**
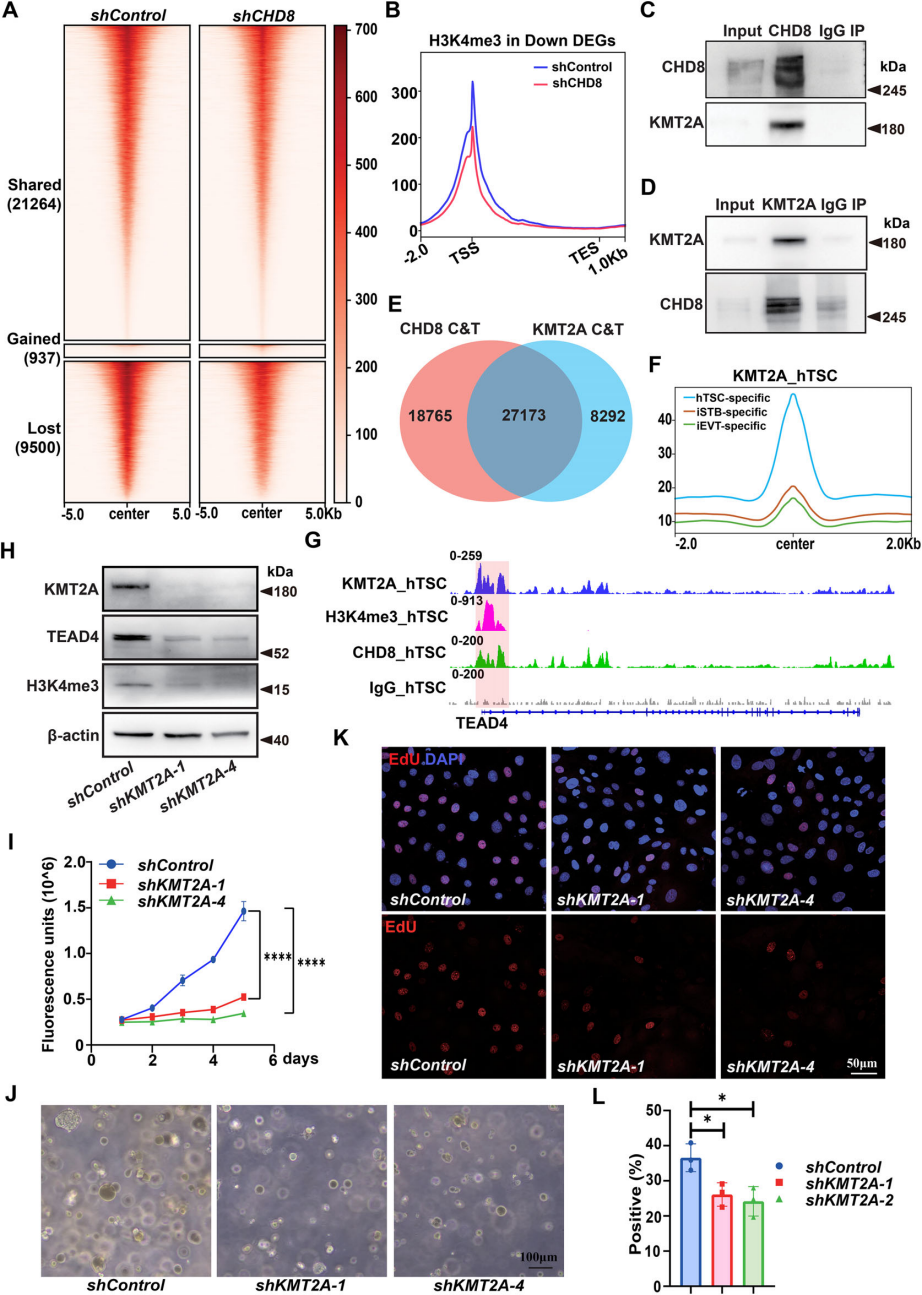
CHD8 is associated with KMT2A‐dependent H3K4me3 at hTSC genes. (A) Heatmap showing the enrichment of H3K4me3 signals at shared, gained, and lost H3K4me3 peaks upon CHD8 depletion in hTSCs. (B) Metaplot showing the enrichment of H3K4me3 signal across down‐regulated genes upon CHD8 depletion in hTSCs. (C) Immunoblots of CHD8 and KMT2A after CHD8 IP in trophoblasts. IgG was used as the negative control. (D) Immunoblots of CHD8 and KMT2A after KMT2A IP in trophoblasts. IgG was used as the negative control. (E) Venn diagram showing the overlap between CHD8 and KMT2A CUT&Tag peaks in hTSCs. (F) Metaplot showing the KMT2A enrichment signal in hTSCs across hTSC‐specific, iEVT‐specific, iSTB‐specific CHD8 peaks. (G) IGV tracks displaying the enrichment of KMT2A, H3K4me3, and CHD8 occupancy at *TEAD4* gene locus in hTSCs. (H) Western blots of KMT2A, TEAD4, and H3K4me3 in *shControl* and *shKMT2A* hTSCs. β‐actin was used as the loading control. (I) PrestoBlue analysis of hTSC cell growth in control and KMT2A‐depleted group (*n* = 3 per group). ****p < 0.0001. (J) Micrographs of organoid formed from *shControl* and *shKMT2A* hTSCs. Scale bar, 100 µm. (K) EdU immunostaining to detect the hTSC proliferation after KMT2A knockdown. Scale bar, 50 µm. (L) The percentages of EdU^+^ hTSCs in *shControl* and *shKMT2A* groups. Biological replicates, *n* = 3. *p < 0.05.

Active chromatin states are reinforced by epigenetic modifications such as H3K4me3, prompting us to examine whether CHD8‐dependent chromatin licensing is coupled to histone methylation. Genome‐wide profiling revealed a substantial loss of H3K4me3 peaks after CHD8 depletion, with only a minor subset of loci gaining the mark (Figure 7A). Integration with ATAC‐seq and CHD8 CUT&Tag showed reduced H3K4me3 signal across CHD8‐bound regions, including CHD8‐bound sites that gained accessibility after CHD8 depletion (Figure S8A). Additionally, we quantified H3K4me3 enrichment across the transcription start sites (TSS) of differentially expressed genes, showing that H3K4me3 was severely reduced at the TSS of down‐regulated genes and modestly reduced at the TSS of up‐regulated and unchanged genes (Figure 7B, Figure S8B). Thus, CHD8 depletion is associated with reduced H3K4me3 at target loci, particularly at genes whose expression depends on CHD8.

To identify candidate CHD8‐interacting proteins that might link CHD8 to H3K4me3, we performed CHD8 immunoprecipitation followed by mass spectrometry in trophoblasts. In addition to known interactors including CHD7 and CTCF [35, 36], we identified MLL‐complex methyltransferase KMT2A as a candidate partner (Figure S8C). Co‐immunoprecipitation confirmed reciprocal CHD8‐KMT2A interactions in trophoblasts (Figure 7C,D). In addition, KMT2A CUT&Tag in hTSCs revealed extensive overlap between CHD8 and KMT2A peaks (Figure 7E). CHD8 hTSC‐specific peaks exhibited higher KMT2A signal intensity than iSTB‐specific and iEVT‐specific peaks (Figure 7F). IGV tracks showed co‐localization of KMT2A, H3K4me3, and CHD8 at the promoters of key TFs, including *TEAD4, GATA3, TFAP2C, WWTR1 and YAP1* (Figure 7G, Figure S8D). These observations suggest a potential functional cooperation between CHD8 and KMT2A in sustaining active H3K4me3 marks at trophoblast regulatory genes.

We next examined whether KMT2A contributes to hTSC maintenance. CHD8 depletion did not alter KMT2A mRNA or protein abundance (Figure S8E,F). Conversely, KMT2A knockdown reduced global H3K4me3 and TEAD4 abundance without affecting CHD8 (Figure 7H; Figure S8G). KMT2A depletion disrupted colony morphology, impaired cell growth, and abolished organoid formation (Figure 7I, J; Figure S8H). EdU incorporation and Ki67 staining confirmed a severe reduction in cycling hTSCs after KMT2A depletion (Figure 7K,L, Figure S8I,J), mirroring the phenotype observed upon CHD8 depletion. Together, these results indicate that CHD8 and KMT2A converge on H3K4me3‐associated transcriptional programs required for hTSC maintenance.

### 2.7 CHD8 Maintains Permissive Chromatin Environment Required for mTSCs

**FIGURE 8:**
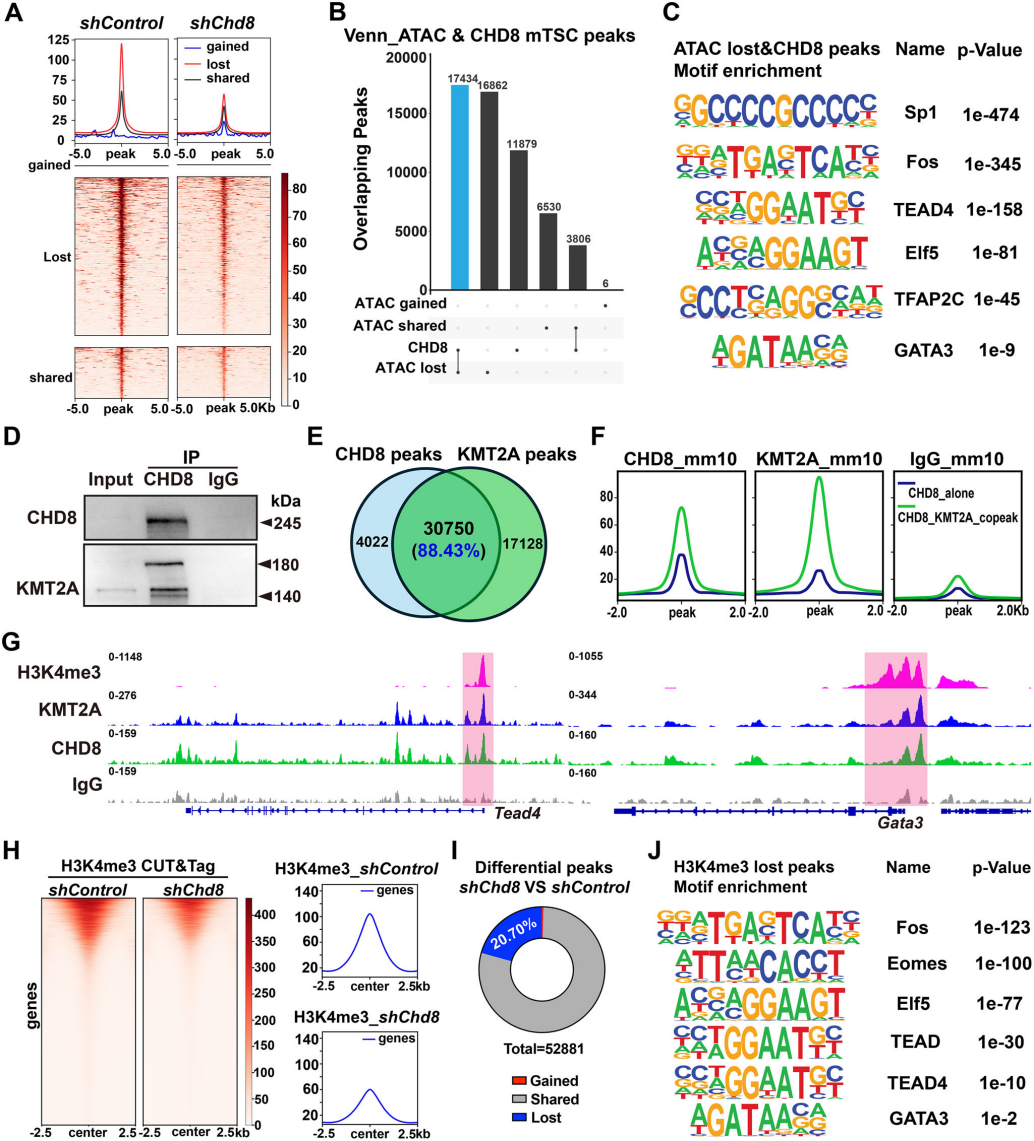
CHD8 maintains permissive chromatin and active H3K4me3 in mTSCs. (A) Heatmap and metaplots of ATAC‐seq signals at shared, gained, and lost accessible regions after *Chd8* depletion in mTSCs. (B) Upset plot depicting the overlap between CHD8 peaks and the shared, gained, and lost ATAC‐seq peaks in mTSCs. (C) Motif enrichment analysis with ATAC‐seq lost&CHD8 peaks in Chd8‐deleted mTSCs using HOMER2. (D) Immunoblots of CHD8 and KMT2A after CHD8 IP in mTSCs. IgG was used as the negative control. (E) Venn diagram showing overlap between CHD8 and KMT2A CUT&Tag peaks in mTSCs. (F) Metaplot showing the intensity of co‐peaks in CHD8, KMT2A, and IgG CUT&Tag signals of mTSCs. (G) IGV tracks displaying the enrichment of KMT2A, H3K4me3, and CHD8 occupancy at *Tead4* and *Gata3* gene locus in mTSCs. (H) Heatmap and metaplots showing the enrichment of H3K4me3 signals in *shControl* and *shChd8* mTSCs. (I) The percentages of lost, gained, and shared H3K4me3 peaks after *Chd8* depletion in mTSCs. (J) Motif enrichment analysis with lost H3K4me3 peaks in *Chd8*‐deleted mTSCs using HOMER2.

To check if CHD8‐mediated chromatin remodeling is conserved in mTSCs, we performed ATAC‐seq in control and *Chd8*‐depleted cells. Chromatin accessibility was globally reduced in *shChd8* mTSCs, with barely increased peaks observed (Figure 8A). Approximately half of the lost accessible regions were bound by CHD8 (Figure 8B). These lost CHD8‐bound regions were enriched for motifs recognized by key trophoblast TFs, including FOS, TEAD4, ELF5, and GATA3 (Figure 8C), supporting that CHD8 governs chromatin accessibility required for key trophoblast TFs bindings in mTSCs as well.

Besides, we also performed CHD8 IP in mTSCs, confirming that KMT2A interacted with CHD8 in mTSCs (Figure 8D). CUT&Tag showed that 88.43% of CHD8 peaks overlapped KMT2A peaks (Figure 8E), and co‐occupied regions displayed stronger signal intensity than regions bound by CHD8 alone (Figure 8F). CHD8, KMT2A, and H3K4me3 were enriched at the promoters of key trophoblast regulators, including *Tead4* and *Gata3* (Figure 8G). H3K4me3 CUT&Tag in control and *Chd*8‐depleted mTSCs further showed a global reduction in H3K4me3 signal (Figure 8H). Differential analysis identified 10 946 lost H3K4me3 peaks (20.70% of all peaks; Figure 8I), which were enriched for motifs associated with EOMES, ELF5, TEAD4, and GATA3 (Figure 8J).

Together, these data indicate that CHD8 is required for maintaining chromatin accessibility and H3K4me3‐associated active chromatin in mTSCs, paralleling its function in hTSCs.

### 2.8 CHD8‐Dependent Chromatin Licensing Sustains Transcriptional Programs Essential for TSC Maintenance

**FIGURE 9:**
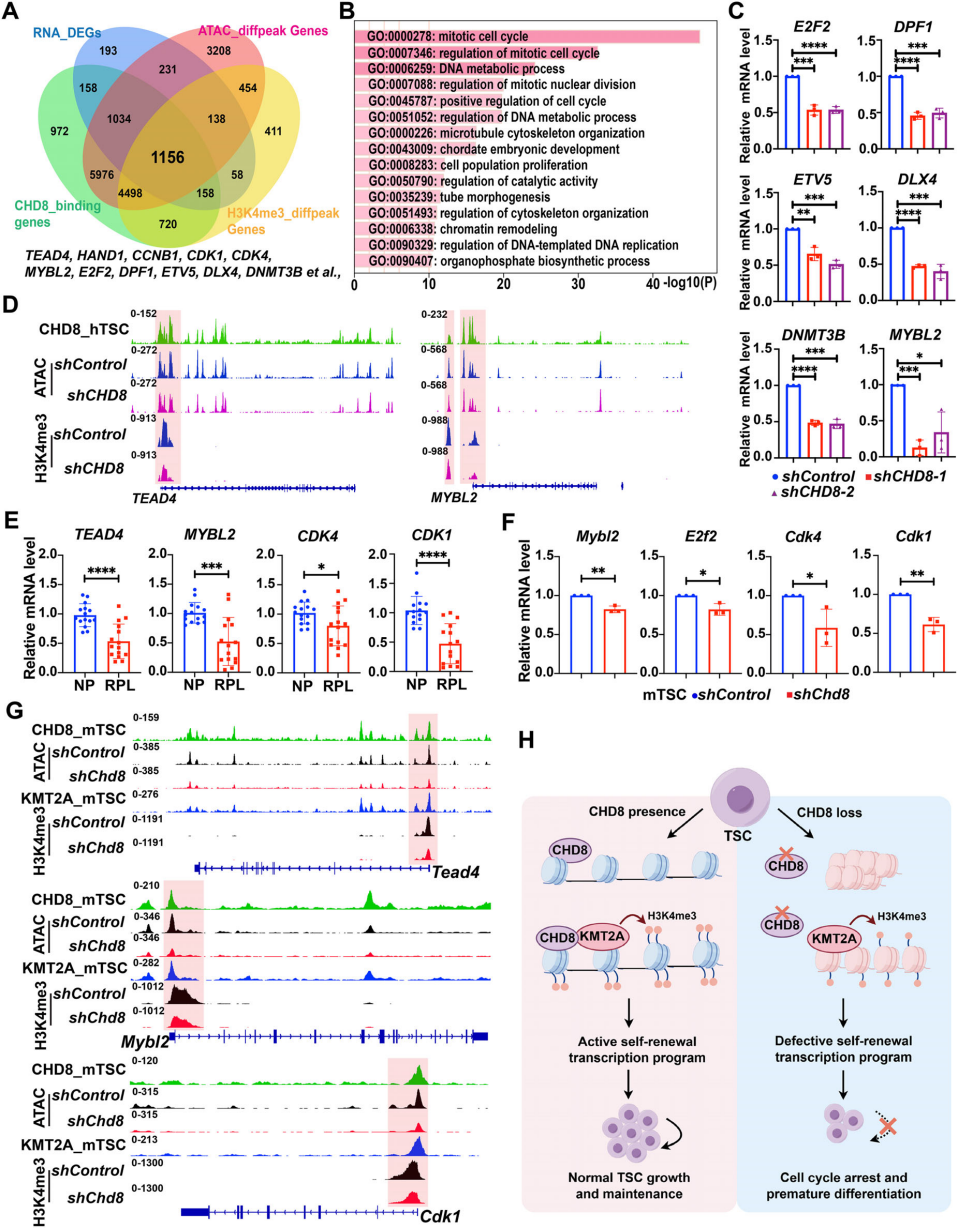
CHD8‐dependent chromatin licensing sustains TSC transcriptional programs. (A) Venn diagram showing the intersection of differentially expressed genes (RNA_DEGs, |Fold change|>1.5), differential H3K4me3 related genes (H3K4me3_diffpeak Genes), differential accessible peaks related genes (ATAC_diffpeak Genes) upon CHD8 depletion, and CHD8 associated genes in hTSCs. (B) Functional enrichment analysis of the 1156 common gene targets from (A), showing significantly enriched biological processes. (C) RT‐qPCR validation of representative candidate targets after CHD8 depletion. The data represent 3 biological replicates. *p < 0.05, **p < 0.01, ***p < 0.001, and ****p < 0.0001. (D) IGV tracks displaying the enrichment of ATAC‐seq signal, H3K4me3, and CHD8 occupancy signals at *TEAD4* and *MYBL2* gene loci in control and *CHD8* depleted hTSCs. (E) RT‐qPCR detection of *TEAD4*, *MYBL2*, *CDK4*, and *CDK1* mRNA in NP and RPL placenta villi. *p < 0.05, ***p < 0.001, and ****p < 0.0001. (F) Relative mRNA level of *Mybl2, Cdk1, E2f2, Cdk4* in *shControl* and *shChd8* mTSCs. *n* = 3. *p < 0.05, **p < 0.01. (G) IGV tracks displaying the enrichment of ATAC‐seq signal, H3K4me3, CHD8, and KMT2A occupancy signals at *Tead4, Mybl2*, and *Cdk1* gene loci in control and *Chd8* depleted mTSCs. (H) Working model of CHD8's role in licensing the chromatin accessibility and H3K4me3 in governing accurate TSC transcription programs.

To identify candidate genes directly regulated by CHD8‐dependent chromatin licensing, we integrated hTSC CHD8 CUT&Tag, RNA‐seq (|fold change|>1.5), ATAC‐seq, and H3K4me3 CUT&Tag data. The intersection yielded more than 1000 candidate direct targets (Figure 9A), including established TSC regulators such as *TEAD4, HAND1, DNMT3B*, and *MYBL2*, as well as core cell‐cycle genes [8, 11, 37, 38]. Further GO analysis confirmed the enrichment of cell‐cycle, DNA metabolic, and chromatin‐remodeling processes (Figure 9B). RT‐qPCR validated the significantly down‐regulation of representative genes after CHD8 depletion (Figure 5D,H, Figure 9C). Concordantly, chromatin accessibility and H3K4me3 signals were reduced at the promoters of *TEAD4*, *MYBL2*, *CDK4*, and *CCNB1* (Figure 9D, Figure S9A). In addition, *TEAD4*, *MYBL2*, *CDK4*, and *CDK1* were also significantly downregulated in RPL placenta villus (Figure 9E). In *Chd8*‐depleted mTSCs, *Tead4, Mybl2, E2f2, Cdk4*, and *Cdk1* were also downregulated (Figures 3G and 9F). Moreover, the chromatin accessibility and H3K4me3 deposition were reduced at their promotors (Figure 9G). These findings identify a conserved set of stemness‐ and proliferation‐associated genes linked to CHD8‐dependent chromatin regulation in TSCs.

To test whether reduced TEAD4 expression alone accounts for the self‐renewal defects caused by CHD8 depletion, we restored TEAD4 expression in CHD8‐depleted hTSCs. Consistent with the persistent impairment of TEAD4 chromatin binding, ectopic TEAD4 expression did not restore normal colony morphology, stemness‐gene expression, or cell‐cycle progression (Figure S9B–E). Thus, restoring TEAD4 abundance alone is insufficient to rescue the CHD8‐depletion phenotype, supporting a broader role for CHD8‐dependent chromatin regulation. Similarly, pharmacological elevation of H3K4me3 with the KDM5 inhibitor CPI‐455 could not rescue the hTSC maintenance defects caused by CHD8 loss (Figure S9F,G).

Together, these data support a working model in which CHD8‐dependent accessibility permits TF binding and is coupled to KMT2A‐associated H3K4me3. In this framework, permissive chromatin architecture represents a prerequisite for productive epigenetic activation and transcriptional maintenance in TSCs.

## 3 Conclusion

In this study, we identify CHD8 as a critical regulator of TSC maintenance and placental development through its role in sustaining permissive chromatin states, including accessible chromatin and active H3K4me3 modification. By integrating human and mouse TSC models, together with trophoblast‐specific genetic perturbation in mice, we demonstrate that CHD8‐dependent chromatin licensing preserves transcriptional programs required for trophoblast function. CHD8 loss reduces chromatin accessibility at key regulatory elements, impairs key TF occupancy, and disrupts stemness‐associated gene expression, ultimately compromising placental development (Figure 9H). These findings establish CHD8 as an essential chromatin regulator in extraembryonic lineages and highlight chromatin remodeling as a fundamental determinant of trophoblast transcriptional competence.

As a chromatin remodeler, CHD8 is well‐known as the negative regulator of P53 signaling, and its loss in neural and hematopoietic progenitors leads to dramatic cell apoptosis [25, 27, 39, 40]. In this study, CHD8 depletion in hTSCs did not substantially increase cell apoptosis, allowing the chromatin and transcriptional consequences of CHD8 loss to be examined without extensive cell death. This context dependence is consistent with observations in human embryonic stem cells, in which CHD8 loss has little effect on apoptosis during self‐renewal or endoderm differentiation but produces a more severe apoptotic phenotype during neuroectoderm differentiation [22]. A recent study further showed that hTSCs are comparatively resilient to genomic instability compared to hPSCs and express relatively low P53 levels together with high levels of autophagy‐related genes, serving as a survival mechanism for chromosomal instability (CIN) in hTSCs [41]. This discrepancy of CIN tolerance in hTSCs may provide a more permissive environment for CHD8 loss and compensate its effect in triggering P53‐mediated apoptosis.

Chromatin architecture dictates the gene regulatory networks (GRNs) that precisely coordinate TSC maintenance and differentiation. It is dynamically reorganized during TSC establishment and lineage commitment [42, 43], and emerging evidence underscores the importance of ATP‐dependent chromatin remodeling complex in trophoblast biology. Brg1, also known as SMARCA4, a key component of the SWI/SNF canonical BAF subcomplex, is essential for mTSC maintenance [7, 17]. It cooperates with EOMES to control TSC chromatin accessibility [16]. The cBAF complex is also required for hTSC differentiation, which is timely controlled by MSX2 [18]. Moreover, dysregulation of nBAF component ARID1A and INO80 complex subunit INO80 are linked to pregnancy complications [44, 45]. These studies implicate the importance of ATP‐dependent chromatin remodeling complex in placentation. Our findings extend this framework by showing that CHD8‐dependent accessibility is required for TSC maintenance in both human and mouse. In particularly, CHD8 supports the occupancy of TEAD4 and GATA3 at their target loci, identifying chromatin accessibility as a major constraint on TF engagement in TSCs.

Our data further connect CHD8‐dependent chromatin accessibility with H3K4me3‐associated promoter activity. CHD8 depletion reduced H3K4me3 at key stemness genes, whereas KMT2A depletion phenocopied several aspects of the hTSC maintenance defect. CHD8 and KMT2A associated in trophoblast cells and extensively co‐occupied hTSC regulatory loci. These observations are consistent with a previous report that CHD8 associates with MLL/KMT2 histone methyltransferase complexes in oligodendrocyte [30]. However, our data do not directly establish that CHD8 recruits KMT2A or define the molecular order among accessibility, KMT2A occupancy, and H3K4me3 deposition. Notably, increasing H3K4me3 levels with a KDM5 inhibitor did not rescue transcription when accessibility was compromised, supporting the conclusion that permissive chromatin architecture is required for productive gene activation.

By integrating CHD8 CUT&Tag, RNA‐seq, ATAC‐seq and H3K4me3 CUT&Tag data in our study, we identified a set of candidate direct targets of CHD8 in hTSCs that included transcription factors, cell‐cycle regulators and chromatin remodeling factors, including *TEAD4, HAND1, MYBL2, CDK1 and DNMT3B* et al., all of which have been proven to be essential for TSC maintenance and placentation [11, 37, 38, 46]. As a conserved master TF required for trophoblast maintenance and differentiation, TEAD4 and its related YAP signaling are well‐studied with the developing of hTSC and organoid models [11, 47, 48, 49, 50]. Our study identified CHD8 as a potential upstream chromatin regulator of TEAD4. CHD8 maintains accessibility at TEAD4‐bound regulatory elements and is associated with H3K4me3 at TEAD4 promoter. Ectopic TEAD4 expression neither restored its occupancy at regions that lost accessibility nor rescued the cellular defects caused by CHD8 depletion, suggesting that the CHD8 dependent accessible chromatin is a prerequisite for TEAD4 driven transcriptional activation in TSC maintenance.

In addition to the defects observed in hTSCs, mTSCs, and the trophoblast‐specific mouse knockout, CHD8 expression was found reduced in RPL villi, suggesting potential clinical relevance. However, the clinical samples from RPL patients were obtained during ongoing miscarriage, in which the fetal demise and placental stress may already have occurred. Reduced CHD8 could therefore be a consequence rather than a cause of placental dysfunction. Due to this limitation, the CHD8 decrease and RPL are more associative, which could not be determined as the primary driver of RPL in humans.

Overall, we identify CHD8 as an essential chromatin remodeler that licenses permissive chromatin environment required for TSC self‐renewal and placental development. By sustaining chromatin accessibility at regulatory elements and coupling this state to KMT2A‐associated H3K4me3, CHD8 supports the transcriptional programs that maintain trophoblast progenitor identity. These findings provide a chromatin‐based framework for understanding how trophoblast gene regulatory networks are stabilized during placental development.

## 4 Experimental Section

### 4.1 Human Samples

This study was approved by the Ethics Committee of the Third Affiliated Hospital of Guangzhou Medical University (Approval number: 2018002) and was conducted in strict accordance with the Declaration of Helsinki. Written informed consent was obtained from all participants. The 1st trimester placenta villous were collected from patients of Recurrent Pregnancy Loss (RPL) and from healthy women who terminated pregnancy at the Third Affiliated Hospital of Guangzhou Medical University. Recurrent pregnancy loss was defined as two or more unexplained miscarriages according to the criteria set by the Practice Committee of the American Society for Reproductive Medicine [51]. Patients with the following conditions were systematically excluded from the study cohort: (1) congenital anomalies of the reproductive system, (2) aberrant karyotypes, (3) endocrine or metabolic disorders, (4) autoimmune disorders, (5) coexisting major illnesses, (6) inadequate pharmaceutical intervention, as well as exposure to hazardous chemicals or radiation. The clinical characteristics of each patient included in this study have been summarized in Table S1. After collection, the villous were rinsed with saline and divided into different parts for fixation and storage in liquid nitrogen.

### 4.2 Animal Experiment

The animal experiments were approved by the Animal Ethical and Welfare Committee of the Guangdong Medical Laboratory Animal Center (Approval number: GY2023‐280) and conducted in accordance with the National Institutes of Health Guide for the Care and Use of Laboratory Animals. The Elf5‐Cre transgenic mice were created as previously reported [52]. *CHD8^f/+^* mice (strain No. T009485) were purchased from GemPharmatech (Nanjing, China) and maintained under specific pathogen‐free (SPF) conditions with a 12‐h light/dark cycle and access to food and water at Guangzhou Medical University Laboratory Animal Center. The primers used for genotyping were listed in Table S2. To achieve trophoblast‐specific CHD8 ablation, *CHD8^f/f^* female mice were crossed with *CHD8^f/+^; Elf5‐Cre* male mice. The morning on which a vaginal plug was observed was designated as embryonic day 0.5 (E0.5).

### 4.3 hTSC Culture and Differentiation

Human trophoblast stem cells (hTSCs) derived from the cytotrophoblast (CTB) of the first‐trimester human placenta (6–9 weeks of gestation) were established and cultured as previously described by Okae et al. [31]. A 35 mm dish was coated with 5 µg/mL collagen IV (Corning, #354233) at 37°C for at least 1 h. hTSC were seeded in the coated 35 mm dish at a density of 0.5–1 × 10^5^ cells and cultured in 2 mL of hTSC medium. The culture medium consisted of Advanced DMEM/F12 (Gibco, #12634028) supplemented with 0.1 mM 2‐mercaptoethanol (Gibco, #21985023), 0.2% FBS (Corning, #35‐081‐CV), 0.5% Penicillin‐Streptomycin (Gibco, #15140122), 0.3% BSA (Sigma–Aldrich, #A9418), 1% ITS‐X supplement (Wako, #094‐06761), 1.5 µg/mL L‐ascorbic acid (Wako, #013‐12061), 50 ng/mL recombinant human epidermal growth factor (rhEGF, PeproTech, #AF‐100‐15), 2 µM CHIR99021 (Wako, #034‐23103), 0.5 µM A83‐01 (Wako, #035‐24113), 1 µM SB431542 (Wako, #031‐24291), 5 µM Y27632 (Wako, #034‐24024) and 0.8 mM VPA (Wako, #227‐01071). Cells were cultured at 37°C in 5% CO2, and the culture medium was replaced every one and a half days.

For EVT induction, hTSC were seeded in a 6‐well plate or 35 mm dish pre‐coated with 1 mg/mL collagen IV at a density of 1 × 10^5^ cells per well and cultured in 2 mL of EVT medium. The EVT culture medium consisted of Advanced DMEM/F12 supplemented with 0.1 mM 2‐mercaptoethanol, 0.5% Penicillin‐Streptomycin, 0.3% BSA, 1% ITS‐X supplement, 100 ng/mL neuregulin (NRG1, R&D, #5898‐NR), 7.5 mM A83‐01(Selleck, #S7692), 2.5 mM Y27632, and 4% KnockOut Serum Replacement (KSR Thermo Fisher, #10828010). Matrigel (Corning, #354234) was added to a final concentration of 2% shortly after suspending the cells in the medium. At day 3, the medium was replaced with EVT medium without NRG1, and Matrigel was added to a final concentration of 0.5%. When the cells reached 80% confluence at day 6, they were dissociated with TrypLE for 10–15 min at 37°C and passaged to a new collagen IV‐coated 6‐well plate at a 1:2 split ratio. The cells were suspended in the EVT medium without NRG1 and KSR, with Matrigel added to a final concentration of 0.5%, and cultured for two additional days.

For induction of STB, hTSC were seeded in a 6‐well plate or 35 mm dish pre‐coated with 2.5 mg/mL collagen IV at a density of 1.25 × 10^5^ cells per well, and cultured in 2 mL of STB medium. The STB culture medium consisted of Advanced DMEM/F12 supplemented with 0.1 mM 2‐mercaptoethanol, 0.5% Penicillin‐Streptomycin, 0.3% BSA, 1% ITS‐X supplement, 2.5 mM Y27632, 2 mM forskolin (Sigma, #F3917‐10MG), and 4% KSR. The medium was replaced at day 3, and the cells were analyzed at day 6.

### 4.4 mTSC Culture

Mouse TSCs were cultured as described previously [33, 53]. Briefly, 35 mm dishes were coated with Matrigel (Corning, #356231) at 37°C for at least 30 min. Cells were cultured in TX medium consisting of Advanced DMEM/F12 supplemented with 1% penicillin‐streptomycin, 2 mM GlutaMAX (Gibco, #35050‐061), nonessential amino acids (Gibco, #11140050), 64 mg/L L‐ascorbic acid 2‐phosphate magnesium (Sigma, #A8960), 0.05% BSA, 1 mM sodium pyruvate (Gibco, #11360‐070), 0.5% B27 (Life Technologies, #17504044), 1% ITS‐X (Wako, #094‐06761), 100 µM 2‐mercaptoethanol (Gibco, #21985023), 25 ng/mL FGF4 (PeproTech, #100‐31), 1 mg/mL heparin (Sigma, #H3149‐500KU), 2 ng/mL TGF‐β (PeproTech, #100‐21), 5 µM Y27632 (Wako, #030‐24021), and 200 nM ZSTK474 (Selleck, #S1072). Medium was changed every other day. Subconfluent cells were passaged with trypsin‐EDTA (Gibco, #25300‐054) for 3 min at 37°C. For differentiation, cells were cultured in TX medium lacking FGF4, heparin, and TGF‐β1 (TX diff) and analyzed on day 6.

### 4.5 CHD8 and KMT2A Knockdown in hTSCs and Chd8 Knockdown in mTSCs

Human *CHD8* and *KMT2A* shRNAs were designed with GPP Web Portal website and were cloned into pLKO.1 backbone. Mouse *Chd8* shRNA were used as previously reported [27]. The sequence of *CHD8*, *KMT2A*, *and mouse Chd8* were listed in Table S3. To generate shRNA lentiviral particles, HEK293T cells were co‐transfected with lentiviral vectors, psPAX2, and pMD2.G at a ratio of 10:7.5:2.5 using jetPRIME Transfection Reagent, following the manufacturer's instructions. After 72 h of culture, the supernatant was collected and concentrated for hTSC or mTSC infection.

### 4.6 TEAD4 Overexpression in hTSCs

TEAD4 cDNA variant1 was cloned into pCDH‐CMV‐EF1a‐copGFP‐T2A‐BSD vector and purchased from Guangzhou IGE Biotechnology Co. The lentiviral particles for overexpression were prepared with the same methods from shRNA preparation. The infected hTSCs were treated with 5 µg/mL Blasticidin solution (Invivogen, #ant‐bl‐1) for two days for positive selection.

### 4.7 Organoid Generation From Human Trophoblast Stem Cells

The organoid formation from hTSCs was conducted as previously reported [11]. Briefly, the hTSCs were harvested and re‐suspended in ice‐cold basic trophoblast organoid medium (b‐TOM). The b‐TOM consisted of Advanced DMEM/F12 supplemented with 10 mM HEPES (Gibco, #15630080), B27 (Gibco, #17504044), N2 (Gibco, #17502048) and 2 mM L‐glutamine (Life Technologies, #25030‐081). After centrifuge, the cells were subsequently re‐suspended in ice‐cold advanced trophoblast organoid medium (aTOM), which is b‐TOM supplemented with 100 ng/mL R‐spondin (PeproTech, #120‐38‐20), 1 µM A83‐01, 100 ng/mL recombinant human epidermal growth factor (rhEGF, PeproTech, #AF‐100‐15), 50 ng/mL recombinant human hepatocyte growth factor (rhHGF, PeproTech, #100‐39), 2.5 µM prostaglandin E2 (Sigma, #P0409‐5MG), 3 µM CHIR99021 and 100 ng/mL Noggin (PeproTech, #120‐10c‐250). Growth factor‐reduced Matrigel (Corning, #356231) was added to the a‐TOM cell suspension to reach a final concentration of 60%. A 50 µl volume of the viscous cell solution containing 1.5 × 10^4^ cells was plated in the center of a 24‐well plate. The organoids were allowed to form for 8 days, with fresh medium being changed every 2 days. Brightfield images were taken to observe the growth of the organoids.

### 4.8 Cell Viability Assay

Cell viability was assessed using the PrestoBlue Cell Viability Reagent (Thermo Fisher Scientific, #A13262). An appropriate number of cells, suspended in 100 µl of complete medium, were seeded into each well of a 96‐well plate. Viability testing was conducted according to the manufacturer's protocol. Changes in cell viability were detected by measuring fluorescence intensity (excitation at 570 nm; emission at 610 nm) using a Multimode microplate reader.

### 4.9 Cell Counting Kit‐8 (CCK‐8) Assay

The cell growth was determined using a CCK‐8 kit (GOONIE, #100‐120). 1 × 10^3^ cells were suspended in 100 µl complete medium and seeded into each well of a 96 well plate. At each designated time point, the culture medium was gently aspirated and replaced with 100 µl fresh media containing 10% CCK‐8 reagent. Following incubation at 37°C for 1 h, the absorbance at 450 nm was recorded using a microplate reader.

### 4.10 Colony Formation Assay

hTSCs were seeded in 6‐well plates precoated with 5 µg/mL Col IV. The depletion of CHD8 in hTSCs was performed as previously described. After the culture medium was removed, the cells were fixed with methanol for 15 min and gently washed three times with PBS. Then the cells were stained with crystal violet solution (Beyotime, #c0121‐100 mL) for 10 min and rinsed with DPBS. After drying in air at room temperature, the culture plate was scanned.

### 4.11 5‐Ethynyl‐2′‐Deoxyuridine (EdU) Assay

Cell proliferation was assessed using the EdU Assay Kit (Beyotime, #C0078) in accordance with the manufacturer's protocol. The cells were incubated with 10 µM pre‐warmed EdU working solution for 30 min. Subsequently, the cells were fixed with 4% paraformaldehyde for 15 min and permeabilized with 0.3% Triton X‐100 for 10 min at room temperature. After removing the permeabilization solution and washing the cells, the Click reaction mixture was added and incubated for 30 min at room temperature in the dark. The nuclei were stained with DAPI. Following additional washing, the samples were mounted with Antifade reagent. The cells were observed using a Nikon laser scanning confocal microscope.

### 4.12 Immunoprecipitation (IP)

IP experiments performed as previously reported [27]. In briefly, HTR8 cells were suspended in Buffer A2 (10 mM HEPES‐KOH pH 7.9, 1.5 mM MgCl2, 10 mM KCl) and incubated on ice for 30 min. Nuclei were spun down and resuspended in Buffer C2 (20 mM Hepes‐KOH pH 7.9, 25% glycerol, 420 mM NaCl, 1.5 mM MgCl2, 0.2 mM EDTA) and incubated on ice for 30 min. For IP, 1mg of nuclear extracts was incubated with 2 ug antibodies overnight at in a buffer containing protease and phosphatase inhibitors. The resulting mixture was centrifuged at 4°C, followed by incubation with 25 µl of prewashed Protein A Dynabeads (#10002D, Life Technologies) for 2 h at 4°C. The proteins were eluted with SDS‐PAGE loading buffer and subjected to Western blot analysis and mass spectrometry analysis (Oebiotech, China).

### 4.13 Immunostaining

For paraffin tissues, sections were deparaffinized, rehydrated, and incubated with sodium citrate buffer (ZSGB‐Bio, #ZLI‐9065) at a high temperature to retrieve the antigens. The sections were then incubated with 3% hydrogen peroxide for 10 min to inactivate endogenous peroxidase activity and blocked with 5% BSA at room temperature for 60 min. After incubation with the indicated primary antibody overnight at 4°C, the sections were incubated with the corresponding secondary antibody at room temperature for 60 min and washed with PBST. The sections were then stained with the DAB working reagent (ZSGB‐Bio, #ZLI‐9019), and counterstained with hematoxylin. Finally, images were obtained under a microscope.

For cultured cells, they were fixed with 4% paraformaldehyde for 15 min, permeabilized with 0.1% Triton X‐100 (Solarbio, #T8200) for 10 min, and blocked with 5% BSA (Solarbio, #A8020) for 1 h at room temperature. The samples were incubated with primary antibodies overnight at 4°C. After washing with PBST (three times, 10 min each), the sample were incubated with secondary antibodies and DAPI (Sigma, #D9542‐1MG). Following additional washing, the samples were mounted with Antifade reagent (Southbiotech, #0100‐01). Confocal fluorescence images were captured using a Nikon laser scanning confocal microscope. The primary antibodies includes: CHD8 (1:200, Proteintech, #29783‐1‐AP), E‐Cadherin (1:200, CST, #3195), CGB (1:200, Abcam, #ab9582), CK7 (1:300, Abcam, #EPR17078), Ki67 (1:200, Invitrogen, #14‐5698‐82), TEAD4 (1:200, Abcam, #ab58310), Cleaved Caspase3 (1:200, CST, 9579S), MMP2 (1:100, Huabio, #HA723308), CDX2 (1:500, Huabio, #ET1605‐4), TFAP2C (1:500, Abcam, #ab218107).

### 4.14 Western Blot Analysis

Cells and tissues were lysed in RIPA buffer (Beyotime, #P0013K) supplemented with protease inhibitors (Sigma, #P8849) and phosphatase inhibitors (Sigma, #11836170001) for 30 min on ice. The lysates were then centrifuged at 14 000 rpm for 15 min at 4°C, and the supernatants were transferred to fresh 1.5 mL microcentrifuge tubes. Protein concentrations were determined using the Bradford assay (Beyotime, #P0006). Protein was mixed with 5X SDS loading buffer and heated at 100°C for 10 min, separated on SDS/polyacrylamide gel (Beyotime) using Tris‐Glycine‐SDS running buffer, and were then transferred onto PVDF membranes (Millipore, ISEQ00010). After blocking, the membrane was incubated with primary antibody overnight at 4°C (CHD8, 1:1000, Proteintech, #29783‐1‐AP; TEAD4, 1:1000, Abcam, #ab58310; KMT2A, 1:1000, CST, #14197S; H3K4me3, 1:1000, CST, #9751S; CGB, 1:1000, Abcepta, #AP13036B; P53, 1:1000, CST, #2527S; β‐actin, 1:1000, CST, #3700S), washed, and then incubated for 1 h with the corresponding horseradish peroxidase (HRP)‐conjugated secondary antibodies. Signals were developed using Immobilon Forte Western HRP ECL substrate (Millipore, #WBLUF0500). Quantification was performed by using ImageJ software.

### 4.15 Quantitative Real‐Time PCR

Total mRNA was extracted from cultured cells and tissue using TRIzol reagent (TaKaRa, #9109). Tissue required additional homogenization and lysis using a tissue homogenizer to ensure complete disruption. First‐strand cDNA was synthesized from 1 µg of mRNA using Reverse Transcriptase (Vazyme, #R333‐01) according to the manufacturer's instructions. Real‐time PCR was performed using SYBR Green master mix (Vazyme, #Q711‐02) on Quant Studio 1 Real‐Time PCR system. Samples were run in at least in technical triplicates, and the results were analyzed using the ΔΔCt method with β‐actin or 18S rRNA as the endogenous control. The sequences of the primers are listed in Table S4.

### 4.16 RNA Sequencing (RNA‐seq) and Data Analysis

For RNA sequencing (RNA‐seq), total RNA extraction and sequencing library preparation were conducted at ShenZhen BGI Genomics Co., Ltd. After quality control (RIN ≥ 7.0, 28S/18S ≥ 1.0), mRNA was enriched using oligo(dT) selection, fragmented, and reverse‐transcribed into cDNA. The constructed libraries were quality‐checked and sequenced following successful quality control. Paired‐end raw sequencing reads were processed with Trim Galore to trim low‐quality reads and remove adapters (with parameters: –trim‐n –paired). Cleaned reads were then mapped to the human hg19 reference genome, guided by the transcript annotations obtained from iGenomes using the RSEM pipeline with the default parameters (v1.1.11) [54]. DeSeq2 (v1.16.1) was applied to differential gene expression analyses with default settings [55], except using biological replicates as covariant. Genes are considered as differentially expressed if they show ≥ 1.5‐fold difference in expressions with adjusted p‐value ≤ 0.05 after correcting for multiple testing by FDR (Benjamini and Hochberg false discovery rate). To assess whether CHD8 depletion affects lineage specific expression, RNA‐Seq reads from Shimizu et al. (hTSC, EVT and STB wild type clones; GSE244252) [32] were downloaded from the National Centre for Biotechnology Information GEO repository and processed as described above.

For functional enrichment analysis, we made use of the functions enrichGO in the package clusterProfiler [56] to carry out Gene Ontology (GO) over‐representation tests and Kyoto Encyclopedia of Genes and Genomes (KEGG) gene set enrichment analysis (GSEA) respectively [56]. For GO analysis, *p*‐values were calculated using hypergeometric distribution, and for GSEA analysis, *p*‐values were calculated based on one million permutations. For both types of analyses, pathways were considered as significant if the FDR‐corrected *p*‐value was ≤ 0.05.

### 4.17 CUT&Tag and Data Analysis

CUT&Tag assays were performed using the Hyperactive Universal CUT&Tag Assay Kit for Illumina Pro (Vazyme, #TD904) according to the manufacturer's protocol. Briefly, approximately 1 × 10^5^ cells were washed and immobilized on 100 µl pre‐activated ConA Beads Pro by gentle rotation at room temperature for 10 min. Primary antibodies (CHD8, Bethyl, #A301‐224; TEAD4, Proteintech, #12418‐1‐AP; KMT2A, Active Motif, #61295; H3K4me3, CST, #9751; GATA3, Huabio, #HA601189; IgG: CST, #3900) were incubated with cells at 4°C overnight, followed by pA/G‐Tnp incubation and DNA fragment. Libraries were constructed and checked for quality by Qubit and Fragment Analyzer by Novogene. Finally, the CUT&Tag libraries were sequenced using Illumina NovaSeq X Plus platform at Novogene. Duplicate samples were included for each group.

For data analysis, paired‐end raw sequencing reads were processed with Trim Galore to trim low‐quality reads and remove adapters (with parameters: *–trim‐n –paired*). Cleaned reads were then mapped to hg19 or mm10 by Bowtie2 (v.2.2.9) with parameters: *–end‐to‐end –very‐sensitive –no‐unal –no‐mixed –no‐discordant ‐I 10 ‐X 500*. PCR duplicates were removed, and only uniquely mapped reads (MAPQ ≥ 10) were kept for downstream analysis using SAMtools (v1.4) [57]. For CHD8 CUT&Tag, to make sure the called peaks maintain high consistency between replicates, the “multiple replicates” mode of the genrich package was used to call peaks, which adopts an approach similar to IDR (https://projecteuclid.org/journals/annals‐of‐applied‐statistics/volume‐5/issue‐3/Measuring‐reproducibility‐of‐high‐throughput‐experiments/10.1214/11‐AOAS466.full) package (Genrich ‐t chd8_replicate1.bam, chd8_replicate2.bam ‐e chrM ‐E hg19‐blacklist.v2.bed ‐q 0.01 ‐o chd8.peak) (https://github.com/jsh58/Genrich). Bigwig coverage on biological replicates merged BAM files were generated using “bamCoverage” command from the package deepTools [58] with parameters: ‐bl hg19‐blacklist.v2.bed ‐bs 20 ‐p 16 –normalizeUsing RPKM ‐b1 IP.bam ‐b2 input.bam –extendReads. “hg19‐blacklist.v2.bed” was obtained from https://github.com/Boyle‐Lab/Blacklist/tree/master/lists/hg19‐blacklist.v2.bed.gz. Homer2 (v4.9.1) was used for motif discovery and enrichment analysis [59]. For motifs at CHD8 occupancy sites and chromatin accessible regions, the search space was defined as a 200 bp window centered at peak summit (findMotifsGenome.pl peakInput.bed hg19 out/ ‐size 200 ‐p 10).

### 4.18 ATAC‐seq and Data Analysis

ATAC‐seq library preparation was conducted using the Hyperactive ATAC‐Seq Library Prep Kit for Illumina (Vazyme, #TD711) according to the manufacturer's guidelines. In briefly, 5 × 10^4^ cells were resuspended in 50 µl of cold lysis buffer and incubated on ice for 10 min to isolate the nuclei. After centrifugation, the nuclei were subjected to a transposition reaction by incubating them with a 50 µl Tn5 transposome/Transposition reaction mix at 37°C for 30 min. The fragmented/transposed DNA was purified using ATAC DNA Extract Beads and amplified for 12 cycles by PCR to construct libraries. Libraries were then checked for quality by Qubit and Fragment Analyzer by Novogene and sequenced using Illumina NovaSeq X Plus platform at Novogene. Duplicate samples were included for each group.

ATAC‐seq data was analyzed similarly as in Hu et al. [60]. In briefly, paired‐end raw sequencing reads were trimmed with Trim Galore, mapped to the hg19 or mm10 reference genome with Bowtie2 (v.2.2.9) with the following parameters: *–N 1 –L 25 –X 2000 –no‐mixed –no‐discordant*. PCR duplicates removed and only uniquely mapped reads (MAPQ ≥ 10) were kept for downstream analysis using SAMtools (v1.4). Differential ATAC peaks were called between corresponding conditions, treating biological duplicates separately. This was performed by using the callpeak function of MACS2 [61] to generate bedgraph files (with parameters: *–B –nomodel –shift ‐100 –extsize 200*), on which the bdgdiff subcommand was applied (with parameters: *–l 200 –g 100*) to call ‘differential peaks’. ChIPSeeker was used to determine overlap with genomic features [62]; in addition, it was also used for peak annotation to nearest genes. Replicate‐merged bedgraph files were generated using the MACS2 callpeak function (with the parameters: –B –SPMR –nomodel –shift ‐100 –extsize 200 –t replicate1.bam replicate2.bam). These bedgraph files were subsequently converted to bigwig format using the bedGraphToBigWig function from Kentin formatics (http://hgdownload.soe.ucsc.edu/downloads.html#source_downloads). Metagene heatmap and profile plots were generated using the deeptools suite. To identify the potential targeted genes for each putative enhancer, the nearest peaks away from the TSS of the gene (within 50 kb) were assigned to this gene as its putative enhancers, as similarly done in Li et al. [63].

### 4.19 Data Availability 

The raw sequence data reported in this paper have been deposited in the Genome Sequence Archive in National Genomics Data Center, China National Center for Bioinformation/Beijing Institute of Genomics, Chinese Academy of Sciences (GSA‐Human: HRA013126, GSA: CRA047683) that are publicly accessible at https://ngdc.cncb.ac.cn/gsa‐human.

### 4.20 Statistical Analysis

Statistical analyses were performed with GraphPad Prism software. Data are presented as the mean ± standard deviation (SD). Differences between two groups were assessed using Student's t‐test. A P value < 0.05 was considered statistically significant. The significance levels are denoted as follows: *p < 0.05, **p < 0.01, ***p < 0.001, and ****p < 0.0001, ns indicates not significant.

## Author Contributions

Z.T., D.C., S.Z., H.W., designed the study, Y.H., Z.H., J.L., Z.T., performed experiments, interpreted data, and generated the figures. L.H., J.L., C.T., Y.L., L.X., S.B., performed experiments. X.F., J.C., L.D., W.S. interpreted data. Z.T., D.C., S.Z., H.W., Y.H., Z.H. wrote manuscript.

## Funding

This work was supported by the The National Natural Science Foundation of China (82271695, 82571925), the National Key Research and Development Program of China (2022YFC2704500, 2022YFC2702501), the National Natural Science Foundation of China (82574430, 82571924, 82371674), the Mobility program of Sino German Center (M‐0586), the Science and Technology Program of Guangzhou (2023A03J0378).

## Conflicts of Interest

The authors declare no conflicts of interest.

## Supporting information




**Supporting File**: advs77823‐sup‐0001‐SuppMat.docx.

## Data Availability

The data that support the findings of this study are available from the corresponding author upon reasonable request.

## References

[1] M. Y. Turco and A. Moffett , “Development of the Human Placenta,” Development 146 (2019): dev163428. 10.1242/dev.163428 31776138

[2] I. Aye , S. Tong , D. S. Charnock‐Jones , and G. C. S. Smith , “The Human Placenta and its Role in Reproductive Outcomes Revisited,” Physiological Reviews 105, no. 4 (2025): 2305–2376. 10.1152/physrev.00039.2024 PMC7617900 40497429

[3] M. Hemberger , C. W. Hanna , and W. Dean , “Mechanisms of Early placental Development in Mouse and Humans,” Nature Reviews Genetics 21, no. 1 (2020): 27–43. 10.1038/s41576-019-0169-4 31534202

[4] H. Papuchova and P. A. Latos , “Transcription Factor Networks in Trophoblast Development,” Cellular and Molecular Life Sciences 79, no. 6 (2022): 337. 10.1007/s00018-022-04363-6 PMC9166831 35657505

[5] C. Du , J. Jiang , Y. Li , et al., “Regulation of Endogenous Retrovirus–Derived Regulatory Elements by GATA2/3 and MSX2 in Human Trophoblast Stem Cells,” Genome Research 33, no. 2 (2023): 197–207. 10.1101/gr.277150.122 PMC10069462 36806146

[6] A. Ghosh , R. Kumar , R. P. Kumar , et al., “The GATA Transcriptional Program Dictates Cell Fate Equilibrium to Establish the Maternal–Fetal Exchange Interface and Fetal Development,” Proceedings of the National Academy of Sciences 121, no. 8 (2024): 2310502121. 10.1073/pnas.2310502121 PMC10895349 38346193

[7] B. L. Kidder and S. Palmer , “Examination of Transcriptional Networks Reveals an Important Role for TCFAP2C, SMARCA4, and EOMES in Trophoblast Stem Cell Maintenance,” Genome Research 20, no. 4 (2010): 458–472. 10.1101/gr.101469.109 PMC2847749 20176728

[8] C. Krendl , D. Shaposhnikov , V. Rishko , et al., “GATA2/3‐TFAP2A/C Transcription Factor Network Couples Human Pluripotent Stem Cell Differentiation to Trophectoderm With Repression of Pluripotency,” Proceedings of the National Academy of Sciences 114, no. 45 (2017): E9579–E9588. 10.1073/pnas.1708341114 PMC5692555 29078328

[9] L. Peng , W. Zhao , C. Xu , et al., “BHLHE40 Cooperates With GATA2/3 to Control Human Syncytiotrophoblast Lineage Differentiation,” Advanced Science 12, no. 44 (2025): 07642. 10.1002/advs.202507642 PMC12667488 40911186

[10] A. Ralston , B. J. Cox , N. Nishioka , et al., “Gata3 Regulates Trophoblast Development Downstream of Tead4 and in Parallel to Cdx2,” Development 137, no. 3 (2010): 395–403. 10.1242/dev.038828 20081188

[11] B. Saha , A. Ganguly , P. Home , et al., “TEAD4 Ensures Postimplantation Development by Promoting Trophoblast Self‐Renewal: An Implication in Early Human Pregnancy Loss,” Proceedings of the National Academy of Sciences 117, no. 30 (2020): 17864–17875. 10.1073/pnas.2002449117 PMC7395512 32669432

[12] L. Xiao , L. Ma , Z. Wang , et al., “Deciphering a Distinct Regulatory Network of TEAD4, CDX2 and GATA3 in Humans for Trophoblast Transition From Embryonic Stem Cells,” Biochimica et Biophysica Acta (BBA) ‐ Molecular Cell Research 1867, no. 9 (2020): 118736. 10.1016/j.bbamcr.2020.118736 32389642

[13] R. Yagi , M. J. Kohn , I. Karavanova , et al., “Transcription Factor TEAD4 Specifies the Trophectoderm Lineage at the Beginning of Mammalian Development,” Development 134, no. 21 (2007): 3827–3836. 10.1242/dev.010223 17913785

[14] S. K. Hota and B. G. Bruneau , “ATP‐Dependent Chromatin Remodeling During Mammalian Development,” Development 143, no. 16 (2016): 2882–2897. 10.1242/dev.128892 PMC5004879 27531948

[15] S. Gourisankar , A. Krokhotin , W. Wenderski , and G. R. Crabtree , “Context‐Specific Functions of Chromatin Remodellers in Development and Disease,” Nature Reviews Genetics 25, no. 5 (2024): 340–361. 10.1038/s41576-023-00666-x PMC11867214 38001317

[16] A. M. Bisia , M. E. Xypolita , E. K. Bikoff , E. J. Robertson , and I. Costello , “Eomesodermin in Conjunction With the BAF Complex Promotes Expansion and Invasion of the Trophectoderm Lineage,” Nature Communications 16, no. 1 (2025): 5079. 10.1038/s41467-025-60417-w PMC12126495 40450029

[17] O. Fedorov , J. Castex , C. Tallant , et al., “Selective Targeting of the BRG/PB1 Bromodomains Impairs Embryonic and Trophoblast Stem Cell Maintenance,” Science Advances 1, no. 10 (2015): 1500723. 10.1126/sciadv.1500723 PMC4681344 26702435

[18] R. Hornbachner , A. Lackner , H. Papuchova , et al., “MSX2 Safeguards Syncytiotrophoblast fate of Human Trophoblast Stem Cells,” Proceedings of the National Academy of Sciences 118, no. 37 (2021): 2105130118. 10.1073/pnas.2105130118 PMC8449346 34507999

[19] Z. Tu and Y. Zheng , “Role of ATP‐Dependent Chromatin Remodelers in Hematopoietic Stem and Progenitor Cell Maintenance,” Current Opinion in Hematology 29, no. 4 (2022): 174–180. 10.1097/MOH.0000000000000710 PMC9257093 35787545

[20] A. Alendar and A. Berns , “Sentinels of Chromatin: Chromodomain Helicase DNA‐Binding Proteins in Development and Disease,” Genes & Development 35, no. 21‐22 (2021): 1403–1430. 10.1101/gad.348897.121 PMC8559672 34725129

[21] R. Bernier , C. Golzio , B. Xiong , et al., “Disruptive CHD8 Mutations Define a Subtype of Autism Early in Development,” Cell 158, no. 2 (2014): 263–276. 10.1016/j.cell.2014.06.017 PMC4136921 24998929

[22] S. Ding , X. Lan , Y. Meng , et al., “CHD8 Safeguards Early Neuroectoderm Differentiation in Human ESCs and Protects From Apoptosis During Neurogenesis,” Cell Death & Disease 12, no. 11 (2021): 981. 10.1038/s41419-021-04292-5 PMC8536677 34686651

[23] L. Gervais , M. van den Beek , M. Josserand , et al., “Stem Cell Proliferation Is Kept in Check by the Chromatin Regulators Kismet/CHD7/CHD8 and Trr/MLL3/4,” Developmental Cell 49, no. 4 (2019): 556–573e6e556. 10.1016/j.devcel.2019.04.033 PMC6547167 31112698

[24] L. Meert , M. Pelicano de Almeida , M. R. Dekker , et al., “A CHD8‐TRRAP Axis Facilitates MYC and E2F Target Gene Regulation in Human Neural Stem Cells,” Iscience 28 (2025): 111978. 10.1016/j.isci.2025.111978 PMC11914185 40104050

[25] A. Nita , Y. Muto , Y. Katayama , A. Matsumoto , M. Nishiyama , and K. I. Nakayama , “The Autism‐Related Protein CHD8 Contributes to the Stemness and Differentiation of Mouse hematopoietic Stem Cells,” Cell Reports 34, no. 5 (2021): 108688. 10.1016/j.celrep.2021.108688 33535054

[26] Z. Tu , C. Fan , A. K. Davis , et al., “Autism‐Associated Chromatin Remodeler CHD8 Regulates Erythroblast Cytokinesis and Fine‐Tunes the Balance of Rho GTPase Signaling,” Cell Reports 40, no. 2 (2022): 111072. 10.1016/j.celrep.2022.111072 PMC9302451 35830790

[27] Z. Tu , C. Wang , A. K. Davis , et al., “The Chromatin Remodeler CHD8 Governs Hematopoietic Stem/Progenitor Survival by Regulating ATM‐Mediated P53 Protein Stability,” Blood 138, no. 3 (2021): 221–233. 10.1182/blood.2020009997 PMC8310427 34292326

[28] M. Nishiyama , K. Nakayama , R. Tsunematsu , T. Tsukiyama , A. Kikuchi , and K. I. Nakayama , “Early Embryonic Death in Mice Lacking the β‐Catenin‐Binding Protein Duplin,” Molecular and Cellular Biology 24, no. 19 (2004): 8386–8394. 10.1128/MCB.24.19.8386-8394.2004 PMC516734 15367660

[29] E. Kerschbamer , M. Arnoldi , T. Tripathi , et al., “CHD8 Suppression Impacts on Histone H3 lysine 36 Trimethylation and Alters RNA Alternative Splicing,” Nucleic Acids Research 50, no. 22 (2022): 12809–12828. 10.1093/nar/gkac1134 PMC9825192 36537238

[30] C. Zhao , C. Dong , M. Frah , et al., “Dual Requirement of CHD8 for Chromatin Landscape Establishment and Histone Methyltransferase Recruitment to Promote CNS Myelination and Repair,” Developmental Cell 45, no. 6 (2018): 753–768.e8. 10.1016/j.devcel.2018.05.022 PMC6063525 29920279

[31] H. Okae , H. Toh , T. Sato , et al., “Derivation of Human Trophoblast Stem Cells,” Cell Stem Cell 22, no. 1 (2018): 50–63e6e56. 10.1016/j.stem.2017.11.004 29249463

[32] T. Shimizu , A. Oike , E. H. Kobayashi , et al., “CRISPR Screening in Human Trophoblast Stem Cells Reveals Both Shared and Distinct Aspects of Human and Mouse Placental Development,” Proceedings of the National Academy of Sciences 120, no. 51 (2023): 2311372120. 10.1073/pnas.2311372120 PMC10742386 38085778

[33] S. Bi , L. Huang , Y. Chen , et al., “KAT8‐Mediated H4K16ac Is Essential for Sustaining Trophoblast Self‐Renewal and Proliferation via Regulating CDX2,” Nature Communications 15, no. 1 (2024): 5602. 10.1038/s41467-024-49930-6 PMC11222414 38961108

[34] W. Yu , J. Tao , H. Cao , et al., “The HAVCR1‐Centric Host Factor Network Drives Zika Virus Vertical Transmission,” Cell Reports 44, no. 4 (2025): 115464. 10.1016/j.celrep.2025.115464 40156834

[35] T. Batsukh , L. Pieper , A. M. Koszucka , et al., “CHD8 Interacts With CHD7, a Protein Which Is Mutated in CHARGE Syndrome,” Human Molecular Genetics 19, no. 14 (2010): 2858–2866. 10.1093/hmg/ddq189 20453063

[36] K. Ishihara , M. Oshimura , and M. Nakao , “CTCF‐Dependent Chromatin Insulator Is Linked to Epigenetic Remodeling,” Molecular Cell 23, no. 5 (2006): 733–742. 10.1016/j.molcel.2006.08.008 16949368

[37] S. Andrews , C. Krueger , M. Mellado‐Lopez , et al., “Mechanisms and Function of de novo DNA Methylation in Placental Development Reveals an Essential Role for DNMT3B,” Nature Communications 14, no. 1 (2023): 371. 10.1038/s41467-023-36019-9 PMC9870994 36690623

[38] H. Zhu , H. Luo , X. Wu , et al., “Vitamin C Inactivates c‐Jun N‐Terminal Kinase to Stabilize Heart and Neural Crest Derivatives Expressed 1 (Hand1) in Regulating Placentation and Maintenance of Pregnancy,” Cellular and Molecular Life Sciences 81, no. 1 (2024): 303. 10.1007/s00018-024-05345-6 PMC11335227 39008099

[39] M. Nishiyama , K. Oshikawa , Y. Tsukada , et al., “CHD8 Suppresses p53‐Mediated Apoptosis Through Histone H1 Recruitment During Early Embryogenesis,” Nature Cell Biology 11, no. 2 (2009): 172–182. 10.1038/ncb1831 PMC3132516 19151705

[40] K. Nitahara , A. Kawamura , Y. Kitamura , K. Kato , S. H. Namekawa , and M. Nishiyama , “Chromatin Remodeler CHD8 Is Required for Spermatogonial Proliferation and Early Meiotic Progression,” Nucleic Acids Research 52, no. 6 (2024): 2995–3010. 10.1093/nar/gkad1256 PMC11014243 38224953

[41] D. Wang , A. Cearlock , K. Lane , et al., “Chromosomal Instability in Human Trophoblast Stem Cells and Placentas,” Nature Communications 16, no. 1 (2025): 3918. 10.1038/s41467-025-59245-9 PMC12032275 40280964

[42] J. R. Ounadjela , K. Zhang , K. J. Kobayashi‐Kirschvink , et al., “Spatial Multiomic Landscape of the Human Placenta at Molecular Resolution,” Nature Medicine 30, no. 12 (2024): 3495–3508. 10.1038/s41591-024-03073-9 PMC12660148 39567716

[43] K. M. Varberg , E. M. Dominguez , B. Koseva , et al., “Extravillous Trophoblast Cell Lineage Development Is Associated With Active Remodeling of the Chromatin Landscape,” Nature Communications 14, no. 1 (2023): 4826. 10.1038/s41467-023-40424-5 PMC10415281 37563143

[44] S. Fantone , R. Mazzucchelli , S. R. Giannubilo , A. Ciavattini , D. Marzioni , and G. Tossetta , “AT‐Rich Interactive Domain 1A Protein Expression in Normal and Pathological Pregnancies Complicated by Preeclampsia,” Histochemistry and Cell Biology 154, no. 3 (2020): 339–346. 10.1007/s00418-020-01892-8 32529396

[45] S. Xian , Y. Zhang , L. Wang , et al., “INO80 Participates in the Pathogenesis of Recurrent Miscarriage by Epigenetically Regulating Trophoblast Migration and Invasion,” Journal of Cellular and Molecular Medicine 25, no. 8 (2021): 3885–3897. 10.1111/jcmm.16322 PMC8051727 33724648

[46] Z. Liu , Y. Tan , W. F. Flynn , et al., “HAND1, Partially Mediated Through Ape‐Specific LTR Binding, Is Essential for Human Extra‐Embryonic Mesenchyme Derivation From iPSCs,” Cell Reports 44, no. 4 (2025): 115568. 10.1016/j.celrep.2025.115568 PMC12082684 40220298

[47] G. Meinhardt , S. Haider , V. Kunihs , et al., “Pivotal Role of the Transcriptional Co‐Activator YAP in Trophoblast Stemness of the Developing Human Placenta,” Proceedings of the National Academy of Sciences 117, no. 24 (2020): 13562–13570. 10.1073/pnas.2002630117 PMC7306800 32482863

[48] G. Meinhardt , H. Waldhäusl , A. I. Lackner , et al., “The Multifaceted Roles of the Transcriptional Coactivator TAZ in Extravillous Trophoblast Development of the Human Placenta,” Proceedings of the National Academy of Sciences 122, no. 16 (2025): 2426385122. 10.1073/pnas.2426385122 PMC12037006 40228123

[49] S. Ray , A. Saha , A. Ghosh , et al., “Hippo Signaling Cofactor, WWTR1, at the Crossroads of Human Trophoblast Progenitor Self‐Renewal and Differentiation,” Proceedings of the National Academy of Sciences 119, no. 36 (2022): 2204069119. 10.1073/pnas.2204069119 PMC9457323 36037374

[50] Y. Yang , W. Jia , Z. Luo , et al., “VGLL1 Cooperates With TEAD4 to Control Human Trophectoderm Lineage Specification,” Nature Communications 15, no. 1 (2024): 583. 10.1038/s41467-024-44780-8 PMC10794710 38233381

[51] E. Dimitriadis , E. Menkhorst , S. Saito , W. H. Kutteh , and J. J. Brosens , “Recurrent Pregnancy Loss,” Nature Reviews Disease Primers 6, no. 1 (2020): 98. 10.1038/s41572-020-00228-z 33303732

[52] S. Kong , G. Liang , Z. Tu , D. Chen , H. Wang , and J. Lu , “Generation of Elf5‐Cre Knockin Mouse Strain for Trophoblast‐Specific Gene Manipulation,” Genesis 56, no. 4 (2018): 23101. 10.1002/dvg.23101 29532590

[53] C. Kubaczka , C. Senner , M. J. Araúzo‐Bravo , et al., “Derivation and Maintenance of Murine Trophoblast Stem Cells Under Defined Conditions,” Stem Cell Reports 2, no. 2 (2014): 232–242. 10.1016/j.stemcr.2013.12.013 PMC3923226 24527396

[54] B. Li and C. N. Dewey , “RSEM: Accurate Transcript Quantification From RNA‐Seq Data With or Without a Reference Genome,” BMC Bioinformatics [Electronic Resource] 12, no. 1 (2011): 323. 10.1186/1471-2105-12-323 PMC3163565 21816040

[55] M. I. Love , W. Huber , and S. Anders , “Moderated Estimation of Fold Change and Dispersion for RNA‐seq Data With DESeq2,” Genome Biology 15, no. 12 (2014): 550. 10.1186/s13059-014-0550-8 PMC4302049 25516281

[56] G. Yu , L. G. Wang , Y. Han , and Q. Y. He , “clusterProfiler: An R Package for Comparing Biological Themes Among Gene Clusters,” OMICS: A Journal of Integrative Biology 16, no. 5 (2012): 284–287. 10.1089/omi.2011.0118 PMC3339379 22455463

[57] H. Li , B. Handsaker , A. Wysoker , et al., “The Sequence Alignment/Map Format and SAMtools,” Bioinformatics 25, no. 16 (2009): 2078–2079. 10.1093/bioinformatics/btp352 PMC2723002 19505943

[58] F. Ramirez , F. Dundar , S. Diehl , B. A. Gruning , and T. Manke , “deepTools: A Flexible Platform for Exploring Deep‐Sequencing Data,” Nucleic Acids Research 42, no. W1 (2014): W187–W191. 10.1093/nar/gku365 PMC4086134 24799436

[59] S. Heinz , C. Benner , N. Spann , et al., “Simple Combinations of Lineage‐Determining Transcription Factors Prime Cis‐Regulatory Elements Required for Macrophage and B Cell Identities,” Molecular Cell 38, no. 4 (2010): 576–589. 10.1016/j.molcel.2010.05.004 PMC2898526 20513432

[60] Z. Hu , D. E. K. Tan , G. Chia , et al., “Maternal Factor NELFA Drives a 2C‐Like State in Mouse Embryonic Stem Cells,” Nature Cell Biology 22, no. 2 (2020): 175–186. 10.1038/s41556-019-0453-8 31932739

[61] Y. Zhang , T. Liu , C. A. Meyer , et al., “Model‐Based Analysis of ChIP‐Seq (MACS),” Genome Biology 9, no. 9 (2008): R137. 10.1186/gb-2008-9-9-r137 PMC2592715 18798982

[62] G. Yu , L. G. Wang , and Q. Y. He , “ChIPseeker: An R/Bioconductor Package for ChIP Peak Annotation, Comparison and Visualization,” Bioinformatics 31, no. 14 (2015): 2382–2383. 10.1093/bioinformatics/btv145 25765347

[63] L. Li , F. Lai , X. Hu , et al., “Multifaceted SOX2‐Chromatin Interaction Underpins Pluripotency Progression in Early Embryos,” Science 382, no. 6676 (2023): adi5516. 10.1126/science.adi5516 38096290

