# Supplementary material for: CHD8‐Dependent Chromatin Licensing Sustains Trophoblast Stem Cell Transcriptional Programs
Source: Adv Sci (Weinh). 2026 Sep 27:e77823. Online ahead of print. doi: 10.1002/advs.77823 (PMC13616375; doi:10.1002/advs.77823)
Supplement: Supplementary file 1 [file ADVS-9999-e77823-s001.docx]

**Supplementary Figures and Legends**

**Supplementary Figure 1. CHD8 binding profiles in hTSC, iSTB and iEVT and its expression in normal and RPL placental villi.** (A) Phase contrast images of hTSCs derived from CTB, induced EVT and STB cells, confirming its growth and differentiation capacity. Scale bar, 100μm. (B) Relative expression of markers in hTSC, differentiated STB and EVT (iSTB and iEVT). Error bars indicate mean ± SD of 3 biological replicates. **P < 0.01, ***P < 0.001, and ****P < 0.0001. (C) Relative *CHD8* mRNA expression in hTSC, iSTB and iEVT. 18S was used as the loading control. Error bars indicate mean ± SD of 3 biological replicates. ns, not significant. (D) Bar plot showing the genomic distribution of CHD8 peaks in hTSC, iEVT and iSTB cells. (E) Functional enrichment analysis of CHD8 hTSC-specific, iSTB-specific, and iEVT-specific peaks associated genes showing the top 5 most enriched KEGG pathways. (F) The cumulative distributions of hTSC, early STB, late STB, and all genes showing the fraction of genes within defined distances (x axis) between their transcription start sites (TSSs) to the nearest hTSC-specific (left panel), and iSTB-specific (right panel) CHD8 peaks. (G) The cumulative distributions of hTSC, early EVT, late EVT, and all genes showing the fraction of genes within defined distances (x axis) between their transcription start sites (TSSs) to the nearest hTSC-specific (left panel), and iEVT-specific (right panel) CHD8 peaks. (H) The percentages of co-binding peaks between hTSC-specific CHD8 peaks and STB-primed factors peaks in hTSC. (I) Metaplots showing the enrichment of ATAC-seq signal in hTSCs across hTSC-specific, iEVT-specific, iSTB-specific CHD8 peaks. (J) IGV tracks displaying the enrichment of ATAC-seq signal and CHD8 occupancy at *SDC1,* *HLA-G* and *CDH1* gene loci in the hTSCs.

**Supplementary Figure 2. Loss of CHD8 blocked the cell cycle progression in hTSCs.** (A) Schematic design of *CHD8* short hairpin RNAs for its knockdown in hTSCs. (B) Ki67 immunostaining in *shControl* and *shCHD8* hTSCs. Scale bar, 50μm. (C) The percentages of Ki67^+^ hTSCs in *shControl* and *shCHD8* groups. Biological replicates, n=3. *P < 0.05, ***P < 0.001. (D) Immunostaining of Cleaved CASPASE3 in *shControl* and *shCHD8* hTSCs. Scale bar, 50μm. (E) Western blots of CHD8 and P53 in control and CHD8 deleted hTSCs. β-actin was used as the loading control. (F) RT-qPCR detection of *CDKN1A*, *BAX* and *BBC3* in *shControl* and *shCHD8* hTSCs. The data represent 3 biological replicates. *P < 0.05, **P < 0.01, ns, not significant.

**Supplementary Figure 3. Conditional deletion of *Chd8* in trophoblast progenitors led to defective placentation and miscarriage.** (A) Immunochemistry of CHD8 in the trophoblasts at E7.5, E9.5 and E11.5 implantation sites. em, embryos. dec, decidual cells. EPC, ectoplacental cone. TGC, trophoblast giant cell. Cp, chorion plate. Jz, junction zone. Lab, labyrinth. Scale bars, 500μm and 300μm. (B) H&E staining of *Chd8 wild type* and *Chd8^f/f^Elf5-Cre* implantation sites at E8.5. Scale bars, 500μm and 100μm. (C) Immunochemistry of Cleaved CASPASE3 in the trophoblasts of *Chd8 wild type* and *Chd8^f/f^Elf5-Cre* implantation sites at E8.5. Scale bar, 300μm. (D) Immunochemistry of CHD8 in *Chd8 wild type* and *Chd8^f/f^Elf5-Cre* implantation sites harvested at E9.5. Scale bar, 300μm. (E) Immunostaining of TEAD4 (red) and E-cadherin (green) in the *Chd8 wild type* and *Chd8^f/f^Elf5-Cre* placentas. Scale bar, 100μm.

**Supplementary Figure 4. RNA-seq analysis of transcripts changes after CHD8 depletion.** (A) PCA plot showing the correlation of replicates among *shControl* and *shCHD8* groups. (B) Volcano plot showing expression changes in genes between CHD8 depleted (*shCHD8*) and control hTSCs. Red and green data points represent genes that are significantly up- and down-regulated, respectively. RNA-Seq triplicates were analyzed for both CHD8 depleted (*shCHD8*) and control hTSCs. (C) The cumulative distributions of genes that are actively expressed (active; FPKM≥5 ), not expressed (FPKM<1) in hTSCs, that are down-regulated (Down), up-regulated (Up) upon CHD8 depletion, and all genes (all) showing the fraction of genes within defined distances (x axis) between their transcription start sites (TSSs) to the nearest EVT-specific CHD8 peaks. (D) Bar plot showing the proportion of hTSC, EVT, and STB lineage genes that are up- (blue) and down-regulated (red) upon CHD8 depletion in hTSCs. (E) Relative mRNA expression of *GATA3*, *TP63, SNAI1, CDK1* in *shControl* and *shCHD8* groups. **P < 0.01, ***P<0.001, ****P < 0.0001, ns, not significant. n=3. (F) Immunostaining of MMP2 in *shControl* and *shCHD8* hTSCs. Scale bar, 20μm. (G) Functional enrichment analysis of up-regulated genes upon CHD8 depletion in hTSCs.

**Supplementary Figure 5. Defective cell cycle process in CHD8-depleted hTSCs.** (A) RT-qPCR confirmation of efficient CHD8 deletion after its knockdown. The data represent 3 biological replicates. ***P<0.001, ****P<0.0001. (B) Co-immunostaining of CGB and EdU in *shControl* and *shCHD8* hTSCs. Scale bar, 50μm. (C) Percentages of EdU^+^CGB^-^ hTSCs in *shControl* and *shCHD8* hTSCs. The data represent 3 biological replicates. **P < 0.01.

**Supplementary Figure 6. CHD8 is required for the maintenance of human trophoblast stem cells.** (A-E) Validation of CHD8 knockdown in an independent hTSC line 2 (#2 hTSC). (A) Immunofluorescence staining of CHD8 in *control* and *shCHD8* hTSCs. Scale bar, 20μm. (B) Phase-contrast images of *control* and *shCHD8* hTSCs. Scale bars, 100μm. (C) CCK-8 assay measuring cell proliferation in *control* and *shCHD8* hTSCs. Biological replicates, n=3. ****P < 0.0001. (D) RT-qPCR analysis of *CHD8*, *TEAD4*, *MYBL2*, cell cycle regulators (*CDK1* and *CDK4*), and differentiation markers (*HLA-G, SDC1,* and *CGB*) in control and *shCHD8* hTSCs. Biological replicates, n=3. ***P<0.001, and ****P<0.0001. (E) Immunofluorescence staining of EdU and CGB in control and *shCHD8* hTSCs. Scale bar, 20μm. (F-J) Validation of CHD8 knockdown in independent hTSC line 4 (#4 hTSC). (F) Immunofluorescence staining of CHD8 in *shControl* and *shCHD8* hTSCs. Scale bar, 20μm. (G) Phase-contrast images of *shControl* and *shCHD8* hTSCs. Scale bar, 100μm. (H) CCK-8 assay measuring cell proliferation in *shControl* and *shCHD8* hTSCs. Biological replicates, n=3. ****P<0.0001. (I) RT-qPCR analysis of CHD8, *TEAD4*, *MYBL2,* cell cycle regulators (*CDK1* and *CDK4*), and differentiation markers (*HLA-G, SDC1*, and *CGB*) in *shControl* and *shCHD8* hTSCs. Biological replicates, n=3. **P<0.01, ***P<0.001, and ****P<0.0001. (J) Immunofluorescence staining of EdU and CGB in *shControl* and *shCHD8* hTSCs. Scale bar, 20μm.

**Supplementary Figure 7. CHD8 knockdown in hTSCs resulted in chromatin accessibility changes restricting GATA3 and TEAD4 binding.** (A) Bar plot showing the genomic distribution of shared, gained, and lost ATAC-seq peaks upon CHD8 depletion in hTSCs. (B and C) The top 10 enriched motifs identified from the gained (B) and lost (C) ATAC-seq peaks upon CHD8 depletion in hTSCs, respectively. P values were calculated from cumulative hypergeometric distribution using the Homer2 package. (D) The cumulative distributions of hTSC, EVT, STB, and all genes showing the fraction of genes within defined distances (x axis) between their transcription start sites (TSSs) to the nearest gained (left panel) and lost ATAC-seq peaks. (E) The cumulative distributions of genes that are actively expressed (active; FPKM≥5), not expressed (FPKM<1) in hTSCs, that are down- (Down) and up-regulated (Up) upon CHD8 depletion, and all genes (all) showing the fraction of genes within defined distances (x-axis) between their transcription start sites (TSSs) to the nearest gained and lost ATAC-seq peaks. (F) The cumulative distributions of genes that are actively expressed (active; FPKM≥5), not expressed (FPKM<1) in hTSCs, that are down-regulated (Down), up-regulated (Up) upon CHD8 depletion, and all genes (all) showing the fraction of genes within defined distances (x axis) between their transcription start sites (TSSs) to the nearest gained ATAC-seq peaks that overlap CHD8 binding sites (Gained_hasCHD8) and that do not overlap CHD8 binding sites (Gained_noCHD8). (G) The top 10 most enriched motifs identified from the gained ATAC-seq peaks upon CHD8 depletion that overlap CHD8 peaks in hTSCs. P values were calculated from cumulative hypergeometric distribution using the Homer2 package. (H) Heatmap showing the enrichment of GATA3 binding signals in control (GATA3_shNC) and CHD8 depleted (GATA3_shCHD8) hTSC cells at gained (top panel), lost (middle panel), and shared (bottom panel) ATAC-seq peaks that overlap CHD8 peaks in hTSCs upon CHD8 depletion. (I) Heatmap showing the enrichment of TEAD4 binding signal in control (TEAD4_shNC), CHD8 depleted (TEAD4_shCHD8), and CHD8 depleted and TEAD4 over-expressed (TEAD4_shCHD8TEAD4OE) hTSCs at gained (top panel), lost (middle panel), and shared (bottom panel) ATAC-seq peaks that overlap CHD8 binding sites in hTSCs upon CHD8 depletion.

**Supplementary Figure 8. CHD8 interacts with KMT2A and is associated with H3K4me3 deposition at hTSC genes.** (A) Metaplots showing the enrichment of H3K4me3 occupancy signals in control and CHD8 depleted hTSCs at gained (left panel), lost (middle panel), and shared (right panel) ATAC-seq peaks that overlap CHD8 binding sites upon CHD8 depletion in hTSCs. (B) Metaplots of H3K4me3 occupancy signals in control and CHD8 depleted hTSCs for down-regulated (Down DEGs; left panel), up-regulated (Up DEGs; middle panel), and not differentially expressed (Non DEGs; right panel) genes upon CHD8 depletion in hTSC cells (*shCHD8* vs. control). (C) Venn diagram of CHD8 IP and IgG IP identified proteins in trophoblast. (D) IGV tracks displaying the enrichment of KMT2A, H3K4me3, and CHD8 occupancy at *GATA3, TFAP2C, WWTR1* and *YAP1* gene locus in hTSCs. (E) Relative expression of *KMT2A* mRNA in CHD8 depleted hTSCs. ns, not significant. (F) Western blot of CHD8, KMT2A and H3K4me3 in *shControl* and *shCHD8* hTSCs. β-actin was used as the loading control. (G) Western blots of CHD8, KMT2A in *shControl* and *shKMT2A* hTSCs. β-actin was used as the loading control. (H) Phase contrast images of *shControl* and *shKMT2A* hTSCs at 72h after lentivirus infection. Scale bar, 100μm. (I) Ki67 immunostaining in *shControl* and *shKMT2A* hTSCs. Scale bar, 50μm. (J) The percentages of Ki67^+^ hTSCs in *shControl* and *shKMT2A* groups. Biological replicates, n=3. *P < 0.05, **P < 0.01.

**Supplementary Figure 9. Overexpression of TEAD4 failed to rescue the self-renewal defects caused by CHD8 depletion in hTSC.** (A) IGV tracks displaying the enrichment of ATAC-seq signal, H3K4me3 and CHD8 occupancy signals at *CDK4* and *CCNB1* gene loci in control and CHD8 depleted hTSC cells. (B) Phase contrast images of *shControl*+empty vector (EV), *shCHD8*+EV, and *shCHD8*+TEAD4 overexpressed hTSCs. Scale bar, 100μm. (C) RT-PCR detection of hTSC genes and CHD8 targets after TEAD4 overexpression in CHD8 deleted hTSCs. The data represent 3 biological replicates. *P < 0.05, **P < 0.01, ***P < 0.001, and ****P < 0.0001, ns, not significant. (D) Ki67 and TEAD4 immunostaining to detect the cell proliferation in *shControl*+empty vector (EV), *shControl*+TEAD4, *shCHD8*+empty vector, and *shCHD8*+TEAD4 hTSCs. Scale bar, 50μm. (E) Percentages of Ki67^+^ cells in *shControl*+empty vector (EV), *shControl*+TEAD4, *shCHD8*+empty vector, and *shCHD8*+TEAD4 hTSCs. **P < 0.01, ns, not significant. (F) Western blots of CHD8, H3K4me3 in *shControl* and *shCHD8* hTSCs treated with CPI-455. β-actin was used as the loading control. (G) Phase contrast images of *shControl* and *shCHD8* hTSCs treated with CPI-455. Scale bar, 200μm.

**Supplementary Tables**

**Supplementary Table 1. The clinical information of the pregnant woman enrolled in the study.**

|  | NP | RPL | P value |
| --- | --- | --- | --- |
| Total sample | 15 | 15 |  |
| Maternal age (year) | 30.60±5.152 | 32.67±3.457 | 0.2076 |
| Gestational age (day) | 54.27±6.053 | 58.40±7.472 | 0.1071 |
| Number of spontaneous abortions | 0 | 2.600±1.121 | ＜0.0001 |

Data are mean ± SD.

|  | **Case No.** | **Maternal age**  **(Year)** | **Gestational age**  **（day）** | **Number of spontaneous abortions** |
| --- | --- | --- | --- | --- |
| NP | 1 | 28 | 55 | 0 |
|  | 2 | 31 | 51 | 0 |
|  | 3 | 31 | 48 | 0 |
|  | 4 | 23 | 62 | 0 |
|  | 5 | 28 | 54 | 0 |
|  | 6 | 35 | 48 | 0 |
|  | 7 | 35 | 52 | 0 |
|  | 8 | 31 | 63 | 0 |
|  | 9 | 38 | 55 | 0 |
|  | 10 | 33 | 49 | 0 |
|  | 11 | 25 | 68 | 0 |
|  | 12 | 22 | 58 | 0 |
|  | 13 | 31 | 52 | 0 |
|  | 14 | 28 | 50 | 0 |
|  | 15 | 40 | 49 | 0 |
| RPL | 1 | 38 | 58 | 3 |
|  | 2 | 36 | 58 | 2 |
|  | 3 | 28 | 66 | 2 |
|  | 4 | 36 | 55 | 2 |
|  | 5 | 34 | 61 | 2 |
|  | 6 | 32 | 46 | 3 |
|  | 7 | 32 | 55 | 2 |
|  | 8 | 28 | 59 | 2 |
|  | 9 | 32 | 65 | 2 |
|  | 10 | 28 | 61 | 4 |
|  | 11 | 33 | 49 | 2 |
|  | 12 | 35 | 46 | 2 |
|  | 13 | 30 | 69 | 6 |
|  | 14 | 30 | 58 | 3 |
|  | 15 | 38 | 70 | 2 |

**Supplementary Table 2. Mouse Genotyping Primers Sequences**

| **Primer Name** | **Sequence (5’-3’)** |
| --- | --- |
| JS03345-Chd8- 5wt-tF1 | GCTAAGGAAGGCTGACTGCAAAC |
| JS03345-Chd8- 5wt-tR1 | TTCTATGATGCACAGGACAGGAG |
| Elf5-F1 | GAATTCTGGAGCGGGTTGAC |
| Elf5-R1 | TAATGGTGCAACGGGTCCTC |
| Cre-98R | TTCTTGCGAACCTCATCACTC |

**Supplementary Table 3. shRNA Sequences**

| **shRNA** | **Forward Primer (5’-3’)** | **Reverse Primer (5’-3’)** |
| --- | --- | --- |
| *shCHD8-1* | CCGGAGCTCCCTTCTGGACAATATACTCGAGTATATTGTCCAGAAGGGAGCTTTTTTG | AATTCAAAAAAGCTCCCTTCTGGACAATATACTCGAGTATATTGTCCAGAAGGGAGCT |
| *shCHD8-2* | CCGGGTAATACCTCATCTGATAATTCTCGAGAATTATCAGATGAGGTATTACTTTTTG | AATTCAAAAAGTAATACCTCATCTGATAATTCTCGAGAATTATCAGATGAGGTATTAC |
| *shKMT2A-1* | CCGGGATTATGACCCTCCAATTAAACTCGAGTTTAATTGGAGGGTCATAATCTTTTTG | AATTCAAAAAGATTATGACCCTCCAATTAAACTCGAGTTTAATTGGAGGGTCATAATC |
| *shKMT2A-4* | CCGGGATTCGAACACCCAGTTATTCCTCGAGGAATAACTGGGTGTTCGAATCTTTTTG | AATTCAAAAAGATTCGAACACCCAGTTATTCCTCGAGGAATAACTGGGTGTTCGAATC |
| *shChd8-1* | CCGGGAATCGAAACGCATCACTTTGCTCGAGCAAAGTGATGCGTTTCGATTCTTTTTG | AATTCAAAAAGAATCGAAACGCATCACTTTGCTCGAGCAAAGTGATGCGTTTCGATTC |

**Supplementary Table 4. RT-qPCR Primer Sequences**

| **Gene** | **Species** | **Forward Primer (5’-3’)** | **Reverse Primer (5’-3’)** |
| --- | --- | --- | --- |
| *CHD8* | Human | CCGGGCATGATCTGCAA | CCGCCGAAGTAGAAGTCATCTT |
| *TEAD4* | Human | CAGGTGGTGGAGAAAGTTGAGA | GTGCTTGAGCTTGTGGATGAAG |
| *KMT2A* | Human | GGATCCACAACTCCAGGCAA | ATTTGGAATGGACCCAGCGA |
| *ACTB* | Human | CTACCTCATGAAGATCCTCACCGA | TTCTCCTTAATGTCACGCACGATT |
| *CDKN1A* | Human | ACCTGTCACTGTCTTGTACCC | CGTTTGGAGTGGTAGAAATCTGTC |
| *BBC3* | Human | GACCTCAACGCACAGTACGA | AAGGGCAGGAGTCCCATGAT |
| **Gene** | **Species** | **Forward Primer (5’-3’)** | **Reverse Primer (5’-3’)** |
| *BAX* | Human | CCCCCGAGAGGTCTTTTTCC | TGTCCAGCCCATGATGGTTC |
| *TP63* | Human | AGAAACGAAGATCCCCAGATGA | CTGTTGCTGTTGCCTGTACGTT |
| *GATA3* | Human | GTCCTGTGCGAACTGTCAGA | TCGGTTTCTGGTCTGGATGC |
| *HLA-G* | Human | CCACCACCCTGTCTTTGACTAT | ACGTCCTGGGTCTGGTCCT |
| *CDK1* | Human | AAACTACAGGTCAAGTGGTAGCC | TCCTGCATAAGCACATCCTGA |
| *SNAI1* | Human | CGAGTGGTTCTTCTGCGCTA | CTGCTGGAAGGTAAACTCTGGA |
| *CGB* | Human | CGTGGCTCTCAGCTGTCAAT | GATGGGCTTGGAAGGCTGG |
| *SDC1* | Human | GCTCATCTTTGCTGTGTGCC | CTCCTGTTTGGTGGGCTTCT |
| *CCNB1* | Human | GCAGCAGGAGCTTTTTGCTT | CCAGGTGCTGCATAACTGGA |
| *CDK4* | Human | CAGTGTACAAGGCCCGTGAT | CAGTCGCCTCAGTAAAGCCA |
| *DLX4* | Human | CAGCACCTAAACCAGCGTTTC | GAGCTTCTTATACTTGGAGCGTT |
| *DNMT3B* | Human | CCCAGCTCTTACCTTACCATCG | GGTCCCCTATTCCAAACTCCT |
| *DPF1* | Human | GGGACAGATTTACACGTACCCC | CACAGTCGATCTTGTACTCGC |
| *E2F2* | Human | GCTCCCCTCTGATCAGCTTC | TCCCCAAGGTCGTAGGAGTC |
| *ETV5* | Human | TCAGCAAGTCCCTTTTATGGTC | GCTCTTCAGAATCGTGAGCCA |
| *HAND1* | Human | CCAAGGATGCACAGTCTGG | AGGAGGAAAACCTTCGTGCTG |
| *MMP2* | Human | TGATGGCATCGCTCAGATCC | GGCCTCGTATACCGCATCAA |
| *MYBL2* | Human | CCCATGAGGAGAACCGCAC | TCAGGTGCTTGGCAATCAGT |
| *18S* | Human | TAGAGGGACAAGTGGCGTTC | CGCTGAGCCAGTCAGTGT |
| *Chd8* | Mouse | GAACCAGTACAGCCAGCGAA | TTGTCCTACGGACCCGTTTC |
| *Tead4* | Mouse | GAAGGCAAGATGTATGGTCGGA | GTTCTTAGCTGCCTGGTCCTT |
| *Cdx2* | Mouse | TCCTGCTGACTGCTTTCTGA | CCCTTCCTGATTTGTGGAGA |
| *Tpbpa* | Mouse | AGAAATGAGTGCCTCCGGTC | CTGTCCATGTTACTGTGGCTGA |
| *Pl1* | Mouse | ACCTGTCACTGTCTTGTACCC | CGTTTGGAGTGGTAGAAATCTGTC |
| *Gata3* | Mouse | CGGGTTCGGATGTAAGTCGA | GTAGAGGTTGCCCCGCAGT |
| *Mybl2* | Mouse | GACCTCCTGGAGCAGAGAGA | CTGGTCTGTGCGGTTAGGAA |
| *Cdk1* | Mouse | ATTGTGTTTTGCCACTCCCG | AGCGTCACTACCTCGTGTG |
| *Cdk4* | Mouse | CTTAGCCGAGCGTAAGGCTG | TCAGGTCCCGGTGAACAATG |
| *E2f2* | Mouse | CTTTGGGAATAGCTCAGAGGCA | GCTCAGGGAGCAACTCTGAA |
| *Actb* | Mouse | GATCAGCAAGCAGGAGTACGA | AAAACGCAGCTCAGTAACAGTC |
